# Suppression of breast cancer-associated bone loss with osteoblast proteomes via Hsp90ab1/moesin-mediated inhibition of TGFβ/FN1/CD44 signaling

**DOI:** 10.7150/thno.66148

**Published:** 2022-01-01

**Authors:** Xun Sun, Kexin Li, Misato Hase, Rongrong Zha, Yan Feng, Bai-Yan Li, Hiroki Yokota

**Affiliations:** 1 Department of Pharmacology, School of Pharmacy, Harbin Medical University, Harbin 150081, China; 2 Department of Biomedical Engineering, Indiana University Purdue University Indianapolis, Indianapolis, IN 46202, USA; 3 Graduate School of Engineering, Mie University, Mie 514, Japan; 4 Indiana Center for Musculoskeletal Health, Indiana University School of Medicine, Indianapolis, IN 46202, USA; 5 Simon Cancer Center, Indiana University School of Medicine, Indianapolis, IN 46202, USA

**Keywords:** breast cancer, osteoblasts, Hsp90ab1, Moesin, CD44

## Abstract

**Background**: Bone is a frequent site of metastases from breast cancer, but existing therapeutic options are not satisfactory. Although osteoblasts have active roles in cancer progression by assisting the vicious bone-destructive cycle, we employed a counterintuitive approach of activating pro-tumorigenic Wnt signaling and examined the paradoxical possibility of developing osteoblast-derived tumor-suppressive, bone-protective secretomes.

**Method**s: Wnt signaling was activated by the overexpression of Lrp5 and β-catenin in osteoblasts as well as a pharmacological agent (BML284), and the therapeutic effects of their conditioned medium (CM) were evaluated using *in vitro* cell cultures, *ex vivo* breast cancer tissues, and a mouse model of osteolysis. To explore the unconventional regulatory mechanism of the action of Wnt-activated osteoblasts, whole-genome proteomics analysis was conducted, followed by immunoprecipitation and gain- and loss-of-function assays.

**Results**: While osteoblasts did not present any innate tumor-suppressing ability, we observed that the overexpression of Lrp5 and β-catenin in Wnt signaling made their CM tumor-suppressive and bone-protective. The growth of breast cancer cells and tissues was inhibited by Lrp5-overexpressing CM (Lrp5 CM), which suppressed mammary tumors and tumor-driven bone destruction in a mouse model. Lrp5 CM also inhibited the differentiation and maturation of bone-resorbing osteoclasts by downregulating NFATc1 and cathepsin K. The overexpression of Lrp5 upregulated osteopontin that enriched Hsp90ab1 (Hsp90 beta) and moesin (MSN) in Lrp5 CM. Hsp90ab1 and MSN are atypical tumor-suppressing proteins since they are multi-tasking, moonlighting proteins that promote tumorigenesis in tumor cells. Importantly, Hsp90ab1 immuno-precipitated latent TGFβ and inactivated TGFβ, whereas MSN interacted with CD44, a cancer stem-cell marker, as well as fibronectin 1, an ECM protein. Furthermore, Hsp90ab1 and MSN downregulated KDM3A that demethylated histones, together with PDL1 that inhibited immune responses.

**Conclusion**: In contrast to inducing tumor-enhancing secretomes and chemoresistance in general by inhibiting varying oncogenic pathways in chemotherapy, this study presented the unexpected outcome of generation tumor-suppressive secretomes by activating the pro-tumorigenic Wnt pathway. The results shed light on the contrasting role of oncogenic signaling in tumor cells and osteoblast-derived secretomes, suggesting a counterintuitive option for the treatment of breast cancer-associated bone metastasis.

## Introduction

Bone is the third most frequent site of metastases from advanced cancers 1, 2. In osteolytic lesions, which are common in breast, lung, and multiple myeloma, cancer cells promote the activity of bone-resorbing osteoclasts 3, whereas osteoblasts orchestrate the development of osteoclasts by secreting RANKL and osteoprotegerin (OPG) 4. RANKL interacts with its receptor, RANK, on the surface of osteoclast progenitors, while OPG is a decoy receptor for RANKL. Osteoclast maturation is stimulated by an increase in the ratio of RANKL to OPG. Osteoblasts also secrete a macrophage colony-stimulating factor (M-CSF) and promote RANKL-stimulated osteoclastogenesis 5, 6. Furthermore, they produce cytokines and growth factors that facilitate the colonization of breast cancer cells 7, 8. For instance, osteoblast-derived TGFβ promotes the progression of tumor cells in the bone 9. Osteoblasts also increase the blood level of CXCL12, which stimulates the proliferation of breast cancer cells 10. Importantly, the development of osteoblasts is driven by Runx2, BMPs, and Wnt signaling, all of which serve as protumorigenic factors 11. Although osteoblasts are bone-forming cells, multiple lines of the evidence above suggest that osteoblasts have active roles in cancer progression and they assist the vicious bone-destructive cycle.

Despite the damaging actions of osteoblasts in the tumor microenvironment, this study examined the possibility of engineering osteoblasts into tumor-suppressing cells. Interestingly, it is reported that a subpopulation of osteoblasts in the tumor-bone microenvironment can retard the growth of breast cancer cells 12. Also, we have previously shown that the overexpression of Lrp5 and β-catenin in Wnt signaling, Akt in PI3K signaling, and Snail in the induction of epithelial-to-mesenchymal transition can grant osteocytes, mesenchymal stem cells (MSCs), and tumor cells tumor-suppressing capabilities 13, 14. We named these genetically engineered osteocytes, MSCs, and tumor cells “induced tumor-suppressing cells” (iTSCs), which generated tumor-suppressive secretomes. Most notably, iTSC-derived conditioned medium (CM) inhibited the growth of mammary tumors and tumor-driven osteolysis in mouse models 15.

Canonical Wnt signaling constitutes an evolutionarily conserved regulatory mechanism for many cellular activities including cell proliferation and development, and Lrp5-mediated Wnt signaling plays a major role in loading-driven bone homeostasis 16. Wnt signaling is also tightly associated with tumorigenesis and in breast cancer, it is activated in over 50% of patients 17. Importantly, Wnt signaling presents a dichotomous role, acting as a tumor promoter in cancer cells and a generator of tumor-suppressive secretomes in osteocyte- and MSC-derived iTSCs 15, 18. Unlike osteocytes or MSCs, however, osteoblasts are the prime contributor to the vicious cycle in the tumor-invaded bone 19. The question herein was whether osteoblasts can be converted into iTSCs by the overexpression of Lrp5, β-catenin, or the application of BML284, a pharmacological activator of Wnt signaling.

The overexpression of Lrp5 was shown to alter the secretomes of osteocytes and MSCs 20. For instance, a tumor suppressor, p53, and an apoptosis inducer selective to tumor cells, Trail, were enriched in MSC-derived iTSC CM 14. In the engineered osteoblasts, we evaluated the role of osteopontin (OPN), an extracellular structural protein, predominantly synthesized by osteoblastic cells 21. OPN is upregulated in a variety of cancers including breast cancer 22, 23. Most notably, however, the result in this study indicated that the role of OPN differed in tumor cells and osteoblasts. Its elevation in tumor cells promoted their tumorigenic behaviors, whereas its upregulation in Lrp5-overexpressing osteoblasts contributed to strengthening tumor-suppressive capabilities. The secretome was enriched with atypical tumor suppressors such as Hsp90ab1 and moesin (MSN). These two proteins, co-regulated with OPN, were previously predicted as tumor suppressors in iTSC secretomes derived from MSCs and osteocytes 14, 15.

To elucidate the mechanism of the anti-tumor action of Hsp90ab1 and MSN, we evaluated their downstream protumorigenic genes such as TGFβ, Runx2, Snail, and MMP9, as well as PDL1 24 that inhibits immune responses and KDM3A 25 that regulates histone methylation. The immunoprecipitation assay revealed that Hsp90ab1 and MSN were co-immunoprecipitated with latent TGFβ and CD44/fibronectin 1 (FN1), respectively, indicating the inhibitory role of Hsp90ab1 in the activation of TGFβ and MSN via the MSN-CD44/FN1 regulatory axis. TGFβ is a potent growth factor that contributes to strengthening the vicious cycle in advanced breast cancer in the bone microenvironment 26. CD44 is a cell-surface adhesion receptor and a cancer stem cell marker 27, 28, while FN1 is an extracellular matrix protein 29. Taken together, the result in this study revealed that the comprehensive anti-tumor effects were obtained with Wnt-activated osteoblasts. Lrp5 CM converted a vicious cycle into a bone-protective beneficial loop, indicating the possibility of developing a unique secretome-based therapeutic strategy for breast cancer and bone metastasis.

## Results

### Tumor-suppressing capability of Lrp5-overexpressing osteoblast-derived CM (Lrp5 CM)

**Figure 1:**
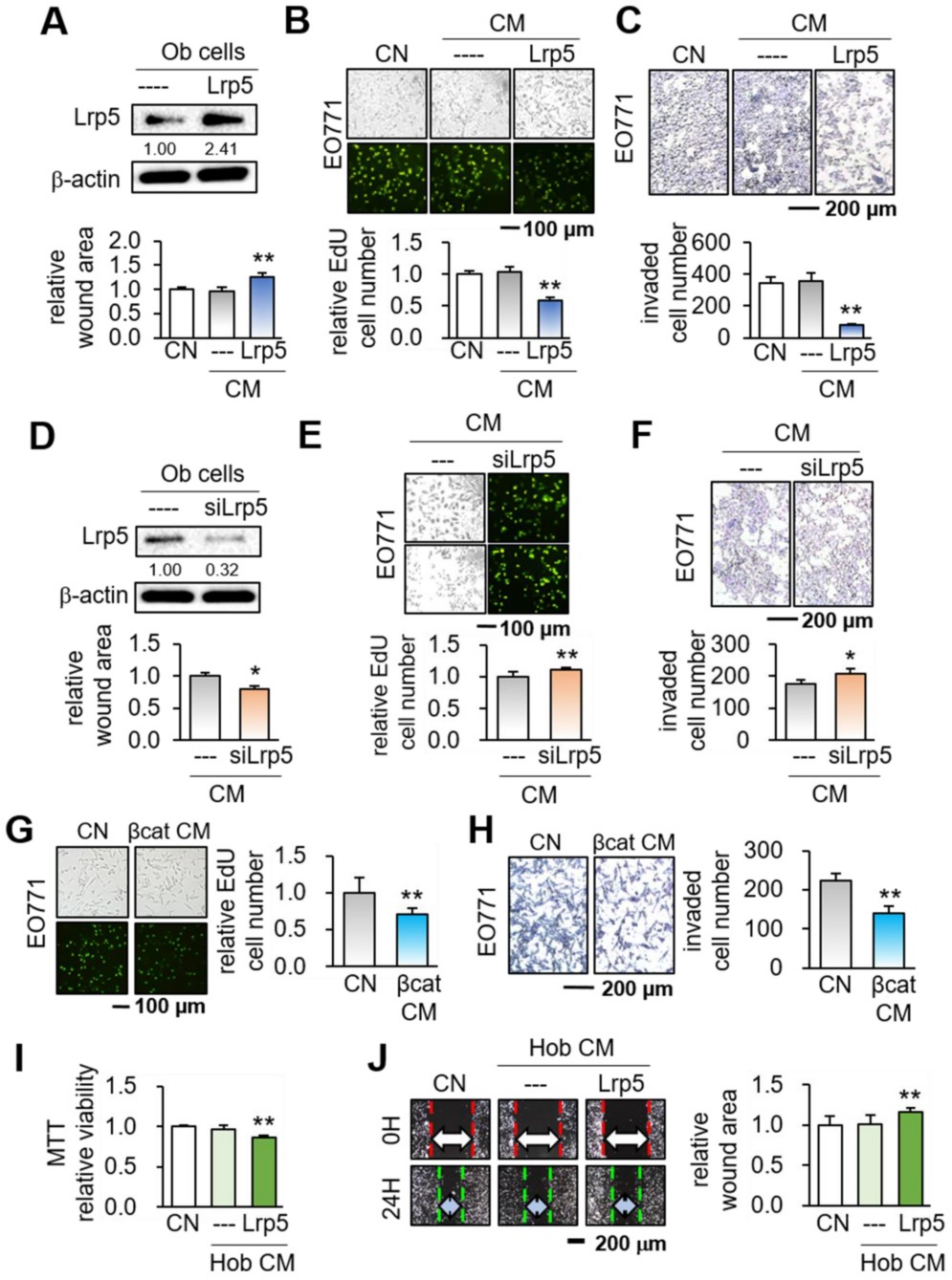
Suppression of the tumorigenic behaviors of EO771 mammary tumor cells by MC3T3 osteoblasts with the overexpression of Lrp5 and β-catenin, and the treatment of BML284. Ob = MC3T3 osteoblasts, Hob = human osteoblasts, CM = osteoblast-derived conditioned medium, siL5 = Lrp5 siRNA, and βcat = β-catenin overexpression. The single and double asterisks indicate p < 0.05 and 0.01, respectively. **(A-C)** Reduction in the scratch-based motility, EdU-based proliferation, and transwell invasion of EO771 mammary tumor cells by Lrp5 CM. **(D-F)** Elevation in the scratch-based motility, EdU-based proliferation, and transwell invasion by Lrp5-silenced CM. **(G-H)** Reduction in the EdU-based proliferation and transwell invasion of EO771 mammary tumor cells by β-catenin Ob CM. **(I-J)** Reduction in the MTT-based proliferation and scratch-based motility of MDA-MB-231 breast cancer cells by Lrp5-overexpressing Hob CM.

We first overexpressed Lrp5 in MC3T3 osteoblasts by transfecting Lrp5 plasmids (Figure 1A). While no detectable changes in tumorigenic behaviors were observed in response to the original osteoblast-derived CM, Lrp5-overexpressing CM (Lrp5 CM) significantly reduced the scratch-based motility in 1 day, EdU-based proliferation in 2 days, and transwell invasion in 2 days of EO771 mammary tumor cells (Figure 1B-C). Consistently, silencing of Lrp5 by RNA interference reversed the responses of EO771 cells (Figure 1D-F). The suppression of the proliferation and invasion was also observed by β-catenin-overexpressing CM, as well as CM derived from BML284-treated osteoblasts (BML CM) (Figure S1A-B). Of note, BML284 is an activator of Wnt signaling. We also employed human osteoblasts (Hob) and showed that Lrp5-overexpressing CM reduced MTT-based cellular viability, scratch-based migration and EdU-based proliferation (Figure 1I-J, Figure S1C).

Besides EO771 mammary tumor cells, we also examined the responses of three other tumor cell lines such as TRAMP murine prostate tumor cells, PC-3 human prostate cancer cells, and MDA-MB-231 human breast cancer cells. The results supported the suppressive effect of Lrp5 CM (Figure S2). Furthermore, β-catenin CM and BML284 CM suppressed the EdU-based proliferation, and transwell invasion of TRAMP and PC-3 prostate tumor cells in 2 days (Figure S3). Immuno-histochemistry revealed that Lrp5 overexpression in osteoblasts elevated the level of β-catenin in their nuclei (Figure S4). Collectively, the result supported the notion that the activation of Wnt signaling by the overexpression of Lrp5 and β-catenin, as well as the treatment with BML284, granted osteoblast-derived CM a tumor-suppressing capability.

### Prevention of bone loss in the tumor-invaded tibia

**Figure 2:**
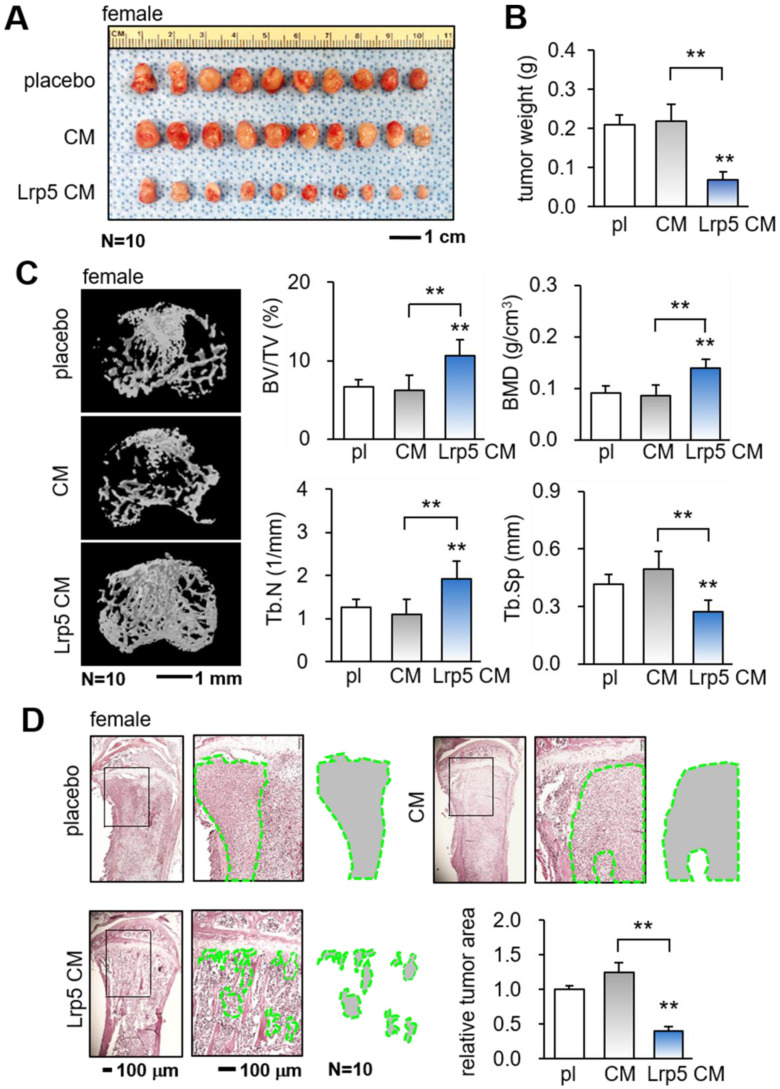
Suppression of the growth of mammary tumors and the protection of tumor-invaded bone by Lrp5 CM. pl = placebo, CM = osteoblast-derived conditioned medium, and Lrp5 = Lrp5 overexpression. The double asterisk indicates p < 0.01. **(A-B)** Reduction in the size and weight of mammary tumors by the daily administration of Lrp5 CM, N = 10. **(C)** Prevention of bone loss in the tumor-invaded tibia by Lrp5 CM. BV/TV = bone volume ratio, BMD = bone mineral density, Tb.N = trabecular number, and Tb.Sp = trabecular separation, N = 10. **(D)** Reduction in the tumor-invaded area (green-outlined region) by the daily administration of Lrp5 CM, N = 10.

The *in vitro* results so far support the tumor-suppressing capability of Lrp5 CM. We next employed a pair of mouse models and evaluated the effect of the daily systemic administration of Lrp5 CM on mammary tumors and tumor-invaded tibiae in female mice. In the mouse model of mammary tumors, the daily intravenous injection of Lrp5 CM from the tail vein for 2 weeks significantly reduced the size and weight of mammary tumors (Figure 2A-B). Furthermore, in the mouse model of tumor-driven osteolysis, the systemic administration of Lrp5 CM inhibited bone loss in the EO771 tumor-invaded proximal tibia (Figure 2C). Lrp5 CM suppressed the reduction in bone volume ratio, bone mineral density, and trabecular number, and it contributed to maintaining trabecular architecture by decreasing trabecular separation. These parameters, derived from microCT images, together with the H&E-stained histological sections demonstrated the reduction in the tumor-invaded area by the administration of Lrp5 CM (Figure 2D). Besides EO771 cells, Lrp5 CM reduced the tumor-invaded area by GFP-labeled MDA-MB-231 breast cancer cells that were inoculated in the tibia of NSG mice (Figure S5A). Consistent with the above result, Lrp5 CM also prevented bone loss in C57BL/6 male mice that received the inoculation of TRAMP prostate tumor cells in the proximal tibia (Figure S5B-C).

### Suppression of tumor invasion *in vivo* and tumor growth *ex vivo*

**Figure 3:**
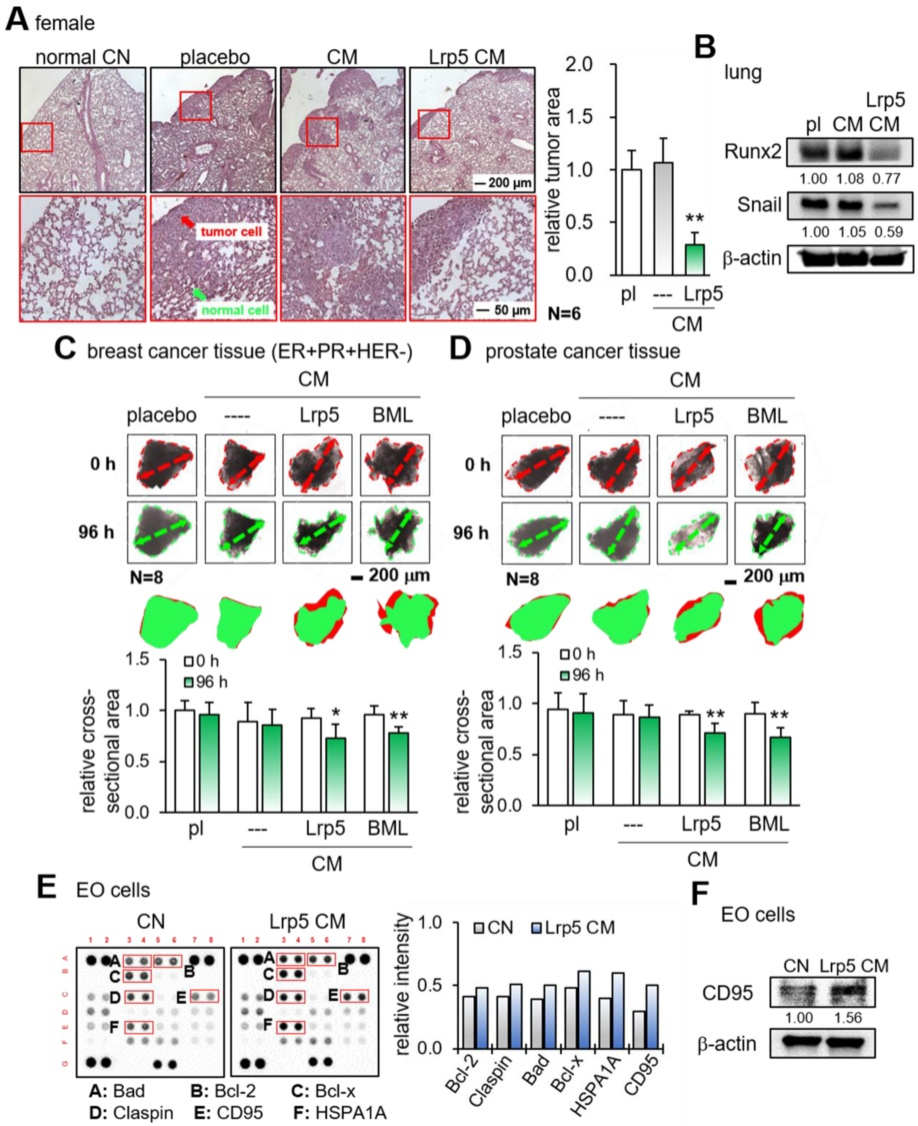
Reduction in tumor invasion to the lung, shrinkage of ex vivo cancer tissue fragments, and elevation of apoptosis-linked proteins in EO771 cells by CM. pl = placebo, Lrp5 = Lrp5 overexpression, CM = osteoblast-derived conditioned medium, and EO = EO771. The single and double asterisks indicate p < 0.05 and 0.01, respectively. **(A)** Reduction in the invasion of EO771 mammary tumor cells into the lung by Lrp5 CM, N = 6. **(B)** Reduction in Runx2 and Snail in the lung by Lrp5 CM. **(C-D)** Shrinkage of ex vivo breast and prostate cancer tissue fragments by Lrp5 CM and BML284-treated CM, N = 8. **(E)** Enrichment of the apoptosis-linked proteins such as Bad, Bcl-2, Bcl-x, Claspin, CD95, and HSPA1A in EO771 breast tumor cells by Lrp5 CM. **(F)** Elevation of CD95 in EO771 breast tumor cells by Lrp5 CM.

We next examined the suppressive effect of Lrp5 CM on the tumor invasion *in vivo* and tumor growth *ex vivo*. First, EO771 mammary tumor cells were introduced as an intra-cardiac injection and their metastasis to the lung was evaluated. In the placebo group, a large number of tumor cells were visible in the lung in 3 weeks, whereas the daily administration of Lrp5 CM for 3 weeks as an intravenous injection significantly reduced the size of tumor-invaded areas in the lung and the expression of oncogenic genes such as Runx2 and Snail in the lung (Figure 3A-B). Consistently, we observed the anti-tumor effects of Lrp5 CM in the lung using GFP-labeled MDA-MB-231 cells that were inoculated as an i.v. injection from the tail vein of NSG female mice (Figure S6). Second, the incubation of freshly isolated breast-cancer-tissue fragments with Lrp5 CM and BML CM significantly reduced the size of tumor fragments in 4 days (Figure 3C). The same anti-tumor response was observed with the cancer fragments, which were derived from prostate cancer tissues (Figure 3D).

To evaluate the effect of Lrp5 CM on the apoptosis induction of tumor cells, we conducted a protein antibody array analysis. The result revealed that apoptosis-linked proteins such as Bad, Bcl-2, Bcl-x, claspin, CD95, and HSPA1A were upregulated in EO771 cells by Lrp5 CM (Figure 3E). Among them, the increase in CD95, a Fas cell surface death receptor, was most significant. Western blotting also showed that the level of CD95 was elevated in response to Lrp5 CM (Figure 3F).

### Stimulation of osteoblast differentiation, inhibition of osteoclast differentiation, and induction of apoptosis

**Figure 4:**
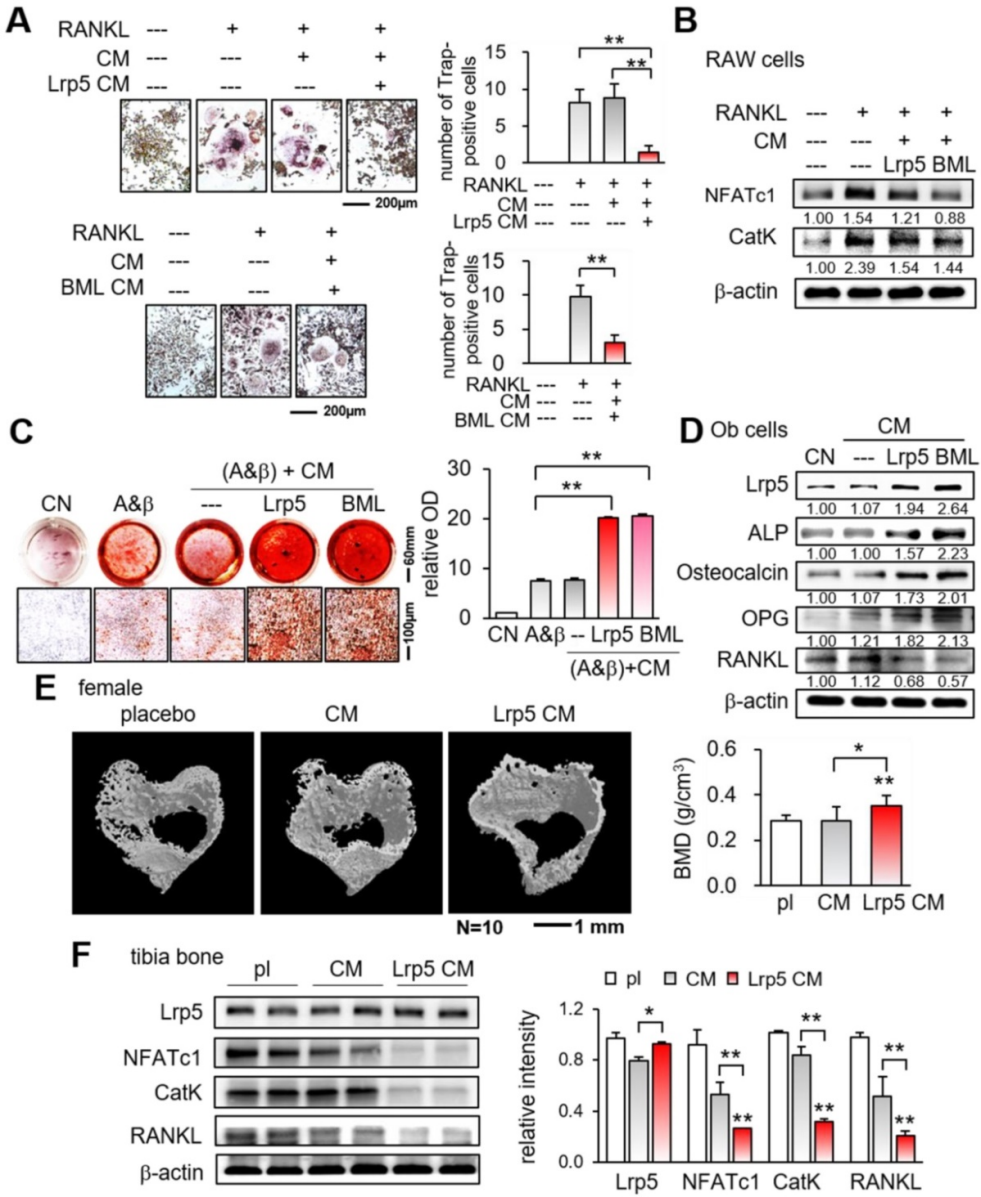
Suppression of osteoclast differentiation and stimulation of osteoblast differentiation by Lrp5 CM and BML284-treated CM. Ob = osteoblasts, CM = osteoblast-derived conditioned medium, A&β = ascorbic acid and β-glycerophosphate, pl = placebo, and Lrp5 = Lrp5 overexpression. The single and double asterisks indicate p < 0.05 and 0.01, respectively. **(A)** Trap staining of RANKL-stimulated RAW264.7 pre-osteoclasts in response to Ob CM, Lrp5 CM, and BML284-treated CM. **(B)** Downregulation of NFATc1 and cathepsin K by Lrp5 CM and BML284-treated CM. **(C)** Enhanced Alizarin-red staining of MC3T3 osteoblasts by Lrp5 CM and BML284-treated CM. **(D)** Elevation of Lrp5, ALP, osteocalcin, and OPG, and the reduction of RANKL by Lrp5 CM and BML284-treated CM in MC3T3 osteoblasts. **(E)** Effect of Lrp5 CM on tibial cortical bone. BMD = bone mineral density, N = 10. **(F)** Downregulation of NFATc1, cathepsin K, and RANKL in the tibia by Lrp5 CM.

We have observed the anti-tumor action of Lrp5 CM, demonstrating that osteoblasts can be converted into iTSCs. We next examined the effect of Lrp5 CM on the differentiation of RAW264.7 pre-osteoclasts and MC3T3 osteoblasts. In response to Lrp5 CM, the number of RANKL-stimulated RAW264.7 cells, which were TRAP-positive and multi-nucleated, was reduced in 5 days (Figure 4A). Furthermore, Lrp5 CM and BML284-treated CM downregulated NFATc1, a master transcription factor for osteoclast differentiation, together with Cathepsin K, a cysteine proteinase largely responsible for the degradation of bone matrix (Figure 4B).

By contrast, the overexpression of Lrp5 in MC3T3 osteoblasts enhanced their staining with Alizarin red and elevated the levels of Lrp5, alkaline phosphatase (ALP), and osteocalcin (Figure S7). Furthermore, the culturing of MC3T3 cells in Lrp5 CM and BML CM increased Alizarin red staining in 4 weeks and elevated the levels of Lrp5, ALP, osteocalcin, and OPG, with a reduced expression level of RANKL (Figure 4C-D). Consistently, in the mouse model of tumor-driven osteolysis microCT imaging revealed that bone mineral density of the cortical bone in the proximal tibia was elevated in the Lrp5 CM group (Figure 4E). Also, the expression levels of NFATc1, cathepsin K, and RANKL were decreased in the tibia by the daily administration of Lrp5 CM (Figure 4F). Collectively, Lrp5 CM and BML284 CM contributed to protecting bone by suppressing the differentiation of bone-resorbing osteoclasts and stimulating that of bone-forming osteoblasts.

### Dichotomous role of OPN in osteoblasts and tumor cells

**Figure 5:**
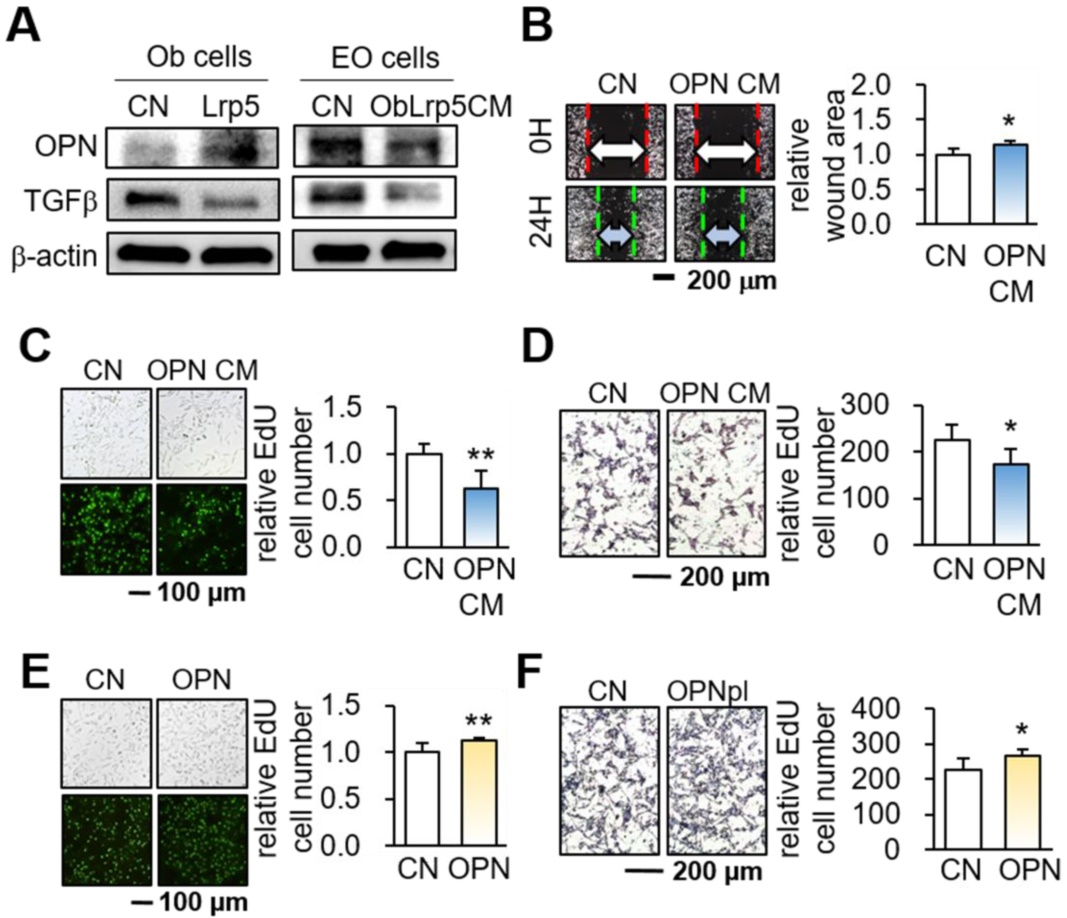
Dichotomous role of osteopontin in osteoblasts and EO771 mammary tumor cells. Lrp5 = Lrp5 overexpression, OPN = osteopontin overexpression, Ob = osteoblasts, EO = EO771, and CM = osteoblast-derived conditioned medium. The single and double asterisks indicate p < 0.05 and 0.01, respectively. **(A)** Osteopontin elevation and TGFβ reduction in Lrp5-overexpressing osteoblasts. The reduction in osteopontin and TGFβ in Lrp5 CM, and Lrp5 CM-treated EO771 mammary tumor cells. **(B-D)** Reduction in the scratch-based motility, EdU-based proliferation, and transwell invasion of EO771 mammary tumor cells by OPN-overexpressing CM. **(E-F)** Stimulation of the EdU-based proliferation and transwell invasion in EO771 cells by the overexpression of OPN.

While OPN is a matrix protein that is mainly synthesized by osteoblasts, its role in tumorigenesis is not completely understood. We observed that the overexpression of Lrp5 in osteoblasts elevated the level of OPN, but Lrp5 CM reduced OPN in EO771 mammary tumor cells (Figure 5A). Of note, the level of TGFβ was downregulated in both Lrp5-overexpressing osteoblasts and Lrp5 CM-treated tumor cells. Importantly, OPN-overexpressing osteoblast-derived CM (OPN CM) suppressed the scratch-based migration, EdU-based proliferation, and transwell invasion of EO771 tumor cells (Figure 5B-D). In agreement with the responses to Lrp5 CM, OPN CM also reduced the levels of Lrp5, Runx2, MMP9, and Snail in EO771 cells (Figure S8A-C). However, OPN-overexpressing EO771 cells promoted their proliferation and invasion (Figure 5E-H) and elevated the levels of Lrp5, Runx2, MMP9, and Snail (Figure S8D). Thus, the results indicated that OPN acted as an anti-tumor agent for generating iTSCs from osteoblasts and a tumor-promoting agent in tumor cells.

### Tumor-suppressing proteins and tumor selectivity

**Figure 6:**
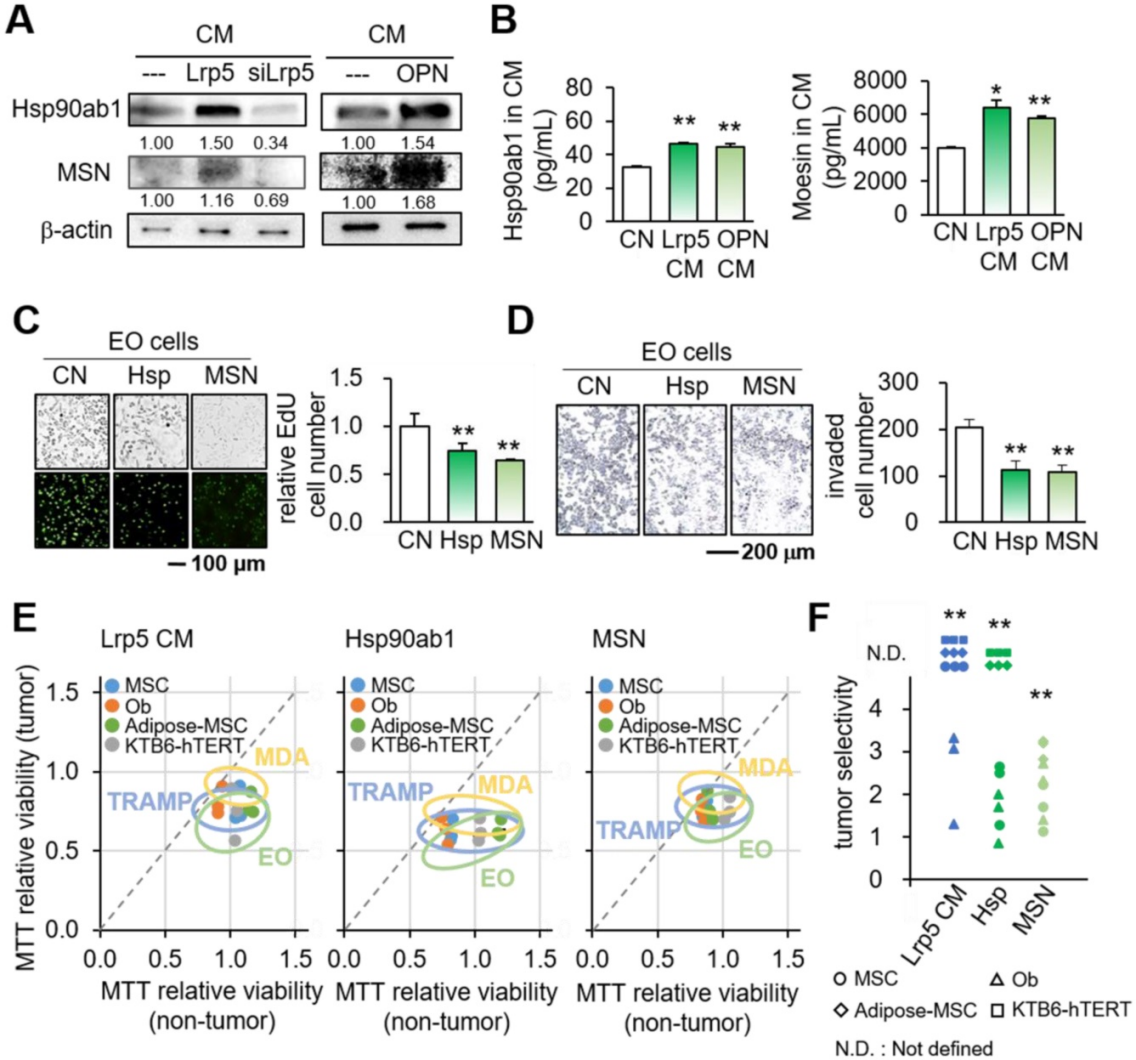
Tumor-suppressing proteins in Lrp5 CM. CN = control, CM = osteoblast-derived conditioned medium, siLrp5 = Lrp5 siRNA, Lrp5 = Lrp5 overexpression, OPN = osteopontin overexpression, Hsp = Hsp90ab1, MSN = moesin, and Ob = osteoblasts. The single and double asterisks indicate p < 0.05 and 0.01, respectively. **(A)** Elevation of Hsp90ab1 and MSN in Lrp5 CM and OPN CM, and the reduction in Lrp5-silenced CM. **(B)** Levels of Hsp90ab1 and moesin in Ob CM and Lrp5 CM. **(C-D)** Reduction in the EdU-based proliferation and transwell invasion of EO771 mammary tumor cells by the application of 5 µg/mL Hsp90ab1 and MSN. **(E-F)** Tumor selectivity of Lrp5 Ob CM, Hsp90ab1, and MSN. Using MTT-based viability, tumor selectivity was defined as the reduction for tumor cells (EO771, MDA-MB-231, and TRAMP cells) to the reduction for non-tumor cells (MSCs, MC3T3, adipose-MSCs, and KTB6-hTERT control epithelial cells). Of note, N.D. (not defined) was used when the metabolic activities of non-tumor cells were stimulated.

We next examined the mechanism of the action of OPN by focusing on Hsp90ab1 and MSN, two atypical tumor-suppressing proteins. These two proteins were selected based on our previous whole-genome proteomics analysis using mass spectrometry 14, 30. In Lrp5 CM, the levels of Hsp90ab1 and MSN were elevated, whereas they were reduced in Lrp5-silenced osteoblast-derived CM (Figure 6A). The ELISA-based levels of Hsp90ab1 and MSN were significantly elevated in Lrp5 CM and OPN CM (Figure 6B). In response to the application of recombinant Hsp90ab1 and MSN, we observed a reduction in the EdU-based proliferation and transwell invasion of EO771 mammary tumor cells (Figure 6C-D). The application of Hsp90ab1 and MSN also downregulated Lrp5, Runx2, MMP9, and Snail in EO771 cells (Figure S8E).

Based on the reduction in MTT-based viability, tumor selectivity was defined as the reduction for tumor cells (EO771, MDA-MB-231, and TRAMP cells) to the reduction for non-tumor cells (MSCs, MC3T3 osteoblasts, adipose-derived MSCs, and KTB6-hTERT epithelial cells). Of note, N.D. (not defined) was used when the viability of non-tumor cells was not inhibited but stimulated. The result showed that the inhibitory effect of Lrp5 CM, Hsp90ab1, and MSN was largely selective to tumor cells than non-tumor cells (Figure 6E-F).

### Diverse effects of Lrp5 CM on histone demethylase and programmed death-ligand

**Figure 7:**
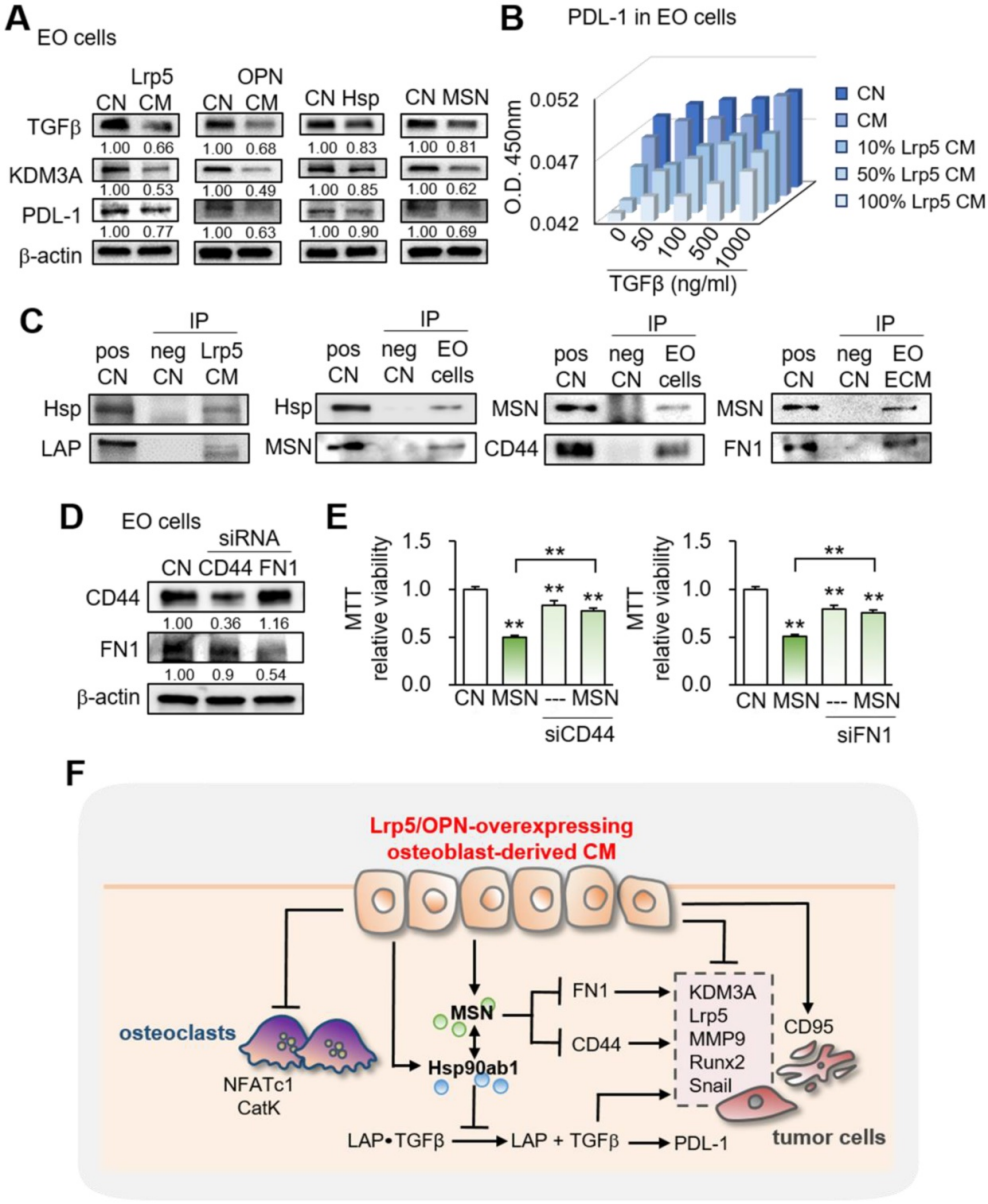
Effects on the expression of TGFβ, KDM3A, and PDL1 and the involvement of CD44 and FN1. CN = control, pos CN = positive control, neg CN = negative control, Lrp5 = Lrp5 overexpression, OPN = osteopontin overexpression, Hsp = Hsp90ab1, MSN = moesin, EO = EO771, ECM = extracellular matrix, CM = osteoblast-derived conditioned medium, siCD44 = CD44 siRNA, and siFN1 = FN1 siRNA. The double asterisks indicate p < 0.01. **(A)** Reduction in TGFβ, KDM3A, and PDL1 by Lrp5 CM, OPN CM, Hsp90ab1, and MSN. **(B)** Level of PDL-1 in EO771 cells by the treatment with TGFβ and Lrp5 CM. **(C)** Co-immunoprecipitation of LAP (latency-associated peptide) by Hsp90ab1 in Lrp5 CM, CD44 by MSN in EO771 cells, and FN1 by MSN in EO771 extracellular matrix. The lane IgG was used as a negative control. **(D-E)** Suppression of MSN-mediated inhibition of the proliferation of EO771 cells by RNA silencing of CD44 and FN1. **(F)** Schematic diagram for the regulatory mechanism of tumor-suppressing action of Lrp5-overexpressing osteoblasts.

Besides the effects on oncogenic genes, we observed that Lrp5 CM reduced the levels of TGFβ, KDM3A, and PDL1, which were linked to matrix degradation, histone methylation, and T-cell-based immune responses, respectively (Figure 7A). In the cell-ELISA assay, the level of PDL1 was elevated by TGFβ and reduced by Lrp5 CM in a dose-dependent manner (Figure 7B, Figure S9).

### Immunoprecipitation of LAP-TGFβ with Hsp90ab1, and CD44/FN1 with MSN

To assess the potential mechanism of tumor-suppressive action of Hsp90ab1 and MSN, we conducted an immunoprecipitation assay. The result revealed that the latent form of TGFβ complex (LAP-TGFβ) was co-immunoprecipitated with Hsp90ab1 in Lrp5 CM, suggesting that Hsp90ab1 can be involved in the activation of TGFβ from its precursor (Figure 7C). In response to recombinant TGFβ proteins, EO771 cells significantly upregulated Lrp5, MMP9, Runx2, and Snail (Figure S10A). Regarding MSN, CD44 and FN1 were co-immunoprecipitated in EO771 cell lysates and extracellular matrix proteins, respectively (Figure 7C). Silencing CD44 and FN1 in EO771 cells and MDA-MB-231 cells downregulated MTT-based viability as well as the expression of Lrp5, MMP9, Runx2, and Snail in EO771 cells, whereas their silencing significantly suppressed MSN-induced tumor inhibition (Figure 7D-E, Figure S10B-D). Taken together, the result indicated the involvement of TGFβ via LAP-TGFβ, cell adhesion via CD44, and extracellular matrix via FN1 in the anti-tumor action of extracellular Hsp90ab1 and MSN.

### Generation of iTSCs from tumor cells and the double-edged role of Lrp5, MSN, and OPN

We have previously shown that iTSCs can be generated from osteocytes and MSCs 14, 20. Lastly, we observed herein that tumor-suppressive secretomes can be induced from tumor cells by the overexpression of Lrp5, MSN, and OPN. Besides osteoblast-derived CM, EO771 tumor cell-derived CM by the overexpression of Lrp5, MSN, or OPN reduced the MTT-based viability of EO771 parent cells (Figure S11A-B). It is noteworthy, however, that EO771 cells with overexpressed Lrp5, MSN, or OPN promoted their MTT-based viability (Figure S11C).

## Discussion

This study presented the tumor-suppressing capability of osteoblasts by the overexpression of Lrp5, as well as β-catenin and the treatment with BML284. Tumorigenic behaviors of breast and prostate cancer cells, such as the proliferation, migration, and invasion, as well as the growth of cancer-tissue fragments, were significantly reduced by the osteoblast-derived secretome. In the mouse model, Lrp5 CM suppressed the progression of mammary tumors and tumor-driven bone loss. Lrp5-overexpressing osteoblasts had an elevated level of OPN, which enriched HSP90ab1 and MSN in CM. Notably, these two proteins acted as extracellular tumor-suppressing proteins while serving as oncogenic proteins in tumor cells. Besides the suppression of tumor progression, Lrp5 CM stimulated osteoblast differentiation and inhibited osteoclast differentiation. The observed inactivation of bone-resorbing osteoclasts contributed to blocking the vicious interplay between tumor cells and osteoclasts and protecting bone loss. Collectively, this study showed that osteoblasts can be converted into iTSCs and Lrp5 CM became an anti-tumor and bone-protective agent (Figure 7F).

The results of this study indicate the differential role of Lrp5 in CMs as extracellular proteins and tumor cells as intracellular proteins. The overexpression of Lrp5 in osteoblasts induced the tumor-suppressive secretome, and its partial silencing reversed the action. By contrast, the elevated expression of Lrp5 was associated with tumorigenic behaviors of tumor cells. Notably, the responses to BML284, an activator of Wnt signaling, were consistent with the dichotomous role of Lrp5-mediated Wnt signaling. The administration of BML284 to osteoblasts converted them into iTSCs, while its application to tumor cells promoted their tumorigenic phenotype. In the bone microenvironment, the result herein suggests the possibility of building a positive feedback loop, as opposed to a vicious cycle, in which Lrp5 CM inhibits the progression of tumor cells and strengthens the anti-tumor action of osteoclasts. Of note, the activation of Lrp5-mediated Wnt signaling is also reported to convert bone-resorbing osteoclasts into bone-protective, tumor-suppressive cells 31.

The previous studies showed that tumor-suppressing proteins, enriched in a group of iTSC CMs, include polyubiquitin C, enolase 1, Hsp90ab1, moesin, Eef2, histone H4, vinculin, and peptidylprolyl isomerase B. Notably, many of these proteins such as Hsp90ab1 and moesin function as a tumor suppressor in the extracellular domain in iTSC CM while a tumor promoter in the intracellular domain 30. While Lrp5 CM, mostly analyzed in this study, contains a spectrum of secretomes, we focused on the role of three moonlighting proteins, OPN, Hsp90ab1, and MSN, for tumor suppression. OPN and MSN are involved in NFκB signaling 32, while Hsp90ab1 is responsive to varying cellular stresses 33. Interestingly, the elevation of these proteins in tumor cells stimulates tumorigenic behaviors. However, the overexpression of Lrp5 in osteoblasts elevated OPN, which increased Hsp90ab1 and MSN in Lrp5 CM. In our previous study 34, a high dose of OPN in osteocytes, which were converted to iTSCs by the overexpression of Lrp5, inhibited tumorigenic genes such as TGFβ and Snail in breast cancer cells. The result in this study with osteoblasts for the role of OPN is consistent with the observed role of Lrp5 and OPN with osteocytes, terminally differentiated osteoblasts. Mechanistically, Hsp90ab1 interacted with latent TGFβ that was inactive without removing the latent associated peptide (LAP) 35. With MSN, two binding partners, CD44 and FN1, co-immunoprecipitated. Silencing each of CD44 and FN1 reduced MSN's anti-tumor effect and their effects were additive. CD44 is a cancer stem cell marker and is known to interact with OPN, FN1, and Ezrin 36-38. Of note, ezrin and MSN belong to the same EZM protein family. FN1 is an ECM protein that interacts with integrin and other ECM proteins such as collagens and its elevated expression is associated with an invasive and metastatic breast cancer phenotype 39. The result indicates that MSN's interaction with CD44 may contribute to repressing breast cancer stem cells. Further investigation of the regulatory mechanism may provide better insight into the precise roles of Hsp90ab1, MSN, CD44, and FN1 in the anti-tumor action of Lrp5 CM.

Chemoresistance and tumor recurrence are two major problems to be solved to treat advanced cancer 40. Emerging evidence shows that the inhibition of oncogenic signaling in chemotherapy induces tumor-promoting secretomes and contributes to building chemoresistance and tumor recurrence. By contrast, we demonstrated in this study that the activation of pro-tumorigenic Wnt signaling generated tumor-suppressive secretomes. The atypical tumor-suppressing proteins interacted with CD44, a breast cancer stem-cell marker, and these interactions drove the elimination of CD44-positive tumor cells. The observed inhibitory actions were selective to tumor cells, compared to non-tumor cells and control epithelial cells. The tumor-suppressing proteins downregulated lysine-specific histone demethylase, as well as PDL1 that is a major target of immunotherapy. Similar to the dichotomous role of Lrp5, we observed a multi-tasking role of OPN, Hsp90ab1, and MSN. Notably, they acted not only as tumor suppressors but also as tumor promoters depending on their cellular locations.

While this study introduced the possibility of the therapeutic use of osteoblast-derived CM, the clinical application of cell-based secretomes is not standard care for cancer patients. Recent clinical trials to test the safety and efficacy of CM-based therapies are directed to the treatments of skin wounds, cerebral palsy, osteoarthritis, and bone regeneration 41. The efficacy and tumor-selectivity may depend on the types of targeted cancers and metastasis sites. For instance, both breast and prostate cancers induce a vicious cycle that leads to a sequence of worsening tumor-driven bone degradation, whereas a major difference is osteoclastic lesions in most breast cancer as opposed to osteoblastic and low-bone-density lesions in prostate cancer. It is worth testing whether anti-tumor iTSC CMs can be generated from other types of cells besides MSCs, osteocytes, and osteoblasts and whether a combinatorial activation of other oncogenic pathways with Wnt signaling may further strengthen anti-tumor actions of iTSCs. It has been shown that iTSCs and their CM can be obtained from T lymphocytes in the peripheral blood 30. While identifying a target to be inhibited and designing inhibitory drugs in chemotherapy is a prime task in the current drug development, identifying an agent to generate iTSCs may offer an alternative possibility.

In conclusion, this study presented the potential usage of osteoblast-derived secretome for the prevention of tumor growth and tumor-driven bone loss in the mouse model. The results indicate the bipartite role of Lrp5, OPN, Hsp90ab1, and MSN in the bone-osteoblast interactions and suggested the possibility of converting a vicious cycle into a beneficial bone-protective loop by uniquely regulating Lrp5-mediated Wnt signaling. The observed moesin-mediated inhibition of CD44 signaling may contribute to removing breast cancer stem cells. Complete or partial secretomes might potentially be useful for developing a novel therapeutic target for breast cancer and bone metastasis.

## Materials and Methods

### Cell culture

EO771 mouse mammary tumor cells (CH3 BioSystems, Amherst, NY, USA) were cultured in DMEM. MDA-MB-231 breast cancer cells (ATCC, Manassas, VA, USA) 42.PC-3 human prostate cancer cells (ATCC) were cultured in RPMI-1640 (Gibco, Carlsbad, CA, USA) 43. RAW264.7 pre-osteoclast cells (ATCC) and MC3T3 osteoblasts (Sigma, St. Louis, MO, USA) were grown in αMEM 44, 45. TRAMP-C2ras prostate tumor cells (ATCC) were cultured in DMEM/F-12. Mouse bone marrow-derived MSCs and human adipose-derived MSCs (Sigma) were grown in MSCBM (Lonza, Basel, Switzerland). KTB6-hTERT human epithelial cells (obtained from Dr. Nakshatri. Indiana University, IN, USA) were cultured in F12 and low glucose DMEM (3:1) with 0.4 µg/mL hydrocortisone (H0888, Sigma), 5 µg/mL insulin (I5500, Sigma) and 20 ng/mL EGF (236-EG-200, R&D Systems, Minneapolis, MN, USA) 46. The culture media was supplemented with 10% fetal bovine serum (FBS) and antibiotics (penicillin and streptomycin), and cells were maintained at 37°C and 5% CO_2_. MC3T3 osteoblasts were treated with 0.2 μM of BML284 (Santa Cruz Biotechnology, Dallas, TX, USA) for 1 day. Tumor cells were treated with 700 ng/mL of recombinant Hsp90ab1 (OPCA05157; Aviva System Biology, San Diego, CA, USA) and MSN (MBS2031729; MyBioSource, San Diego, CA, USA).

CM was generated without FBS and subjected to low-speed centrifugation at 2,000 rpm for 10 min. The cell-free supernatants were centrifuged at 4,000 rpm for 10 min and subjected to filtration through a 0.22-μm polyethersulfone membrane (Sigma). The supernatants were further centrifuged at 10,000 × g for 30 min at 4 °C to remove remaining cell debris and at 100,000 × g (Type 90 Ti Rotor, Beckman, Brea, CA, USA) overnight at 4 °C to remove exosomes. For *in vivo* experiments, CM was condensed ~10 fold using an ultracentrifuge filter (3 kD cut off; Amicon, Sigma) and the final protein concentration was ~1 mg/mL.

### EdU assay, transwell invasion assay, and scratch assay

Using the procedure previously described 47, cellular proliferation was examined using a fluorescence-based cell proliferation kit (Click-iT™ EdU Alexa Fluor™ 488 Imaging Kit; Thermo-Fisher, Waltham, MA, USA). A transwell invasion assay was conducted to detect cell motility, and a wound-healing scratch assay was utilized to evaluate 2-dimensional cell migratory behavior 48.

### Western blot analysis and ELISA assay

Western blot analysis was conducted using the procedure previously described 49. We used antibodies against, β-catenin, Lrp5, Runx2, Snail, TGFβ, MSN, PDL1, CD44 (Cell Signaling, Danvers, MA, USA), MMP9, NFATc1, cathepsin K, OPN, alkaline phosphatase, FN1 (Santa Cruz Biotechnology), CD95, RANKL, OPG (Invitrogen, Carlsbad, CA, USA), Hsp90ab1, Osteocalcin (Abcam, Cambridge, UK), KDM3A (Proteintech, Rosemont, IL, USA), and β-actin (Sigma). The levels of Hsp90ab1 and MSN in Lrp5 CM were determined using ELISA kits (MBS7700502 and MBS2124159; MyBioSource). The level of PDL-1 in EO771 cells, which were treated with 0.05, 0.1, 0.5, and 1 μg/mL of TGFβ and Lrp5 CM, was determined using a cell ELISA kit (PI62200; Invitrogen).

### Immunoprecipitation

Immunoprecipitation was conducted with an immunoprecipitation starter pack kit (Cytiva, Marlborough, MA, USA), using the procedure the manufacture provided. In brief, 20 µL of protein A sepharose was washed twice with PBS and incubated with 2 µg of rabbit-raised antibodies for Hsp90ab1 or MSN. In parallel, rabbit IgG was prepared for negative control. We employed three kinds of protein samples, including Lrp5 CM for Hsp90ab1, as well as EO771 cell lysate and EO771 extracellular matrix proteins for MSN. The antibody-cross-linked beads were incubated overnight with 600 µl protein samples on a shaker. The beads were collected by centrifugation, washed three times with PBS, and resuspended for Western blotting. The protein samples before the immunoprecipitation were used as positive controls. Western blotting was conducted using antibodies against Hsp90ab1, MSN, latency-associated peptide (LAP) for TGFβ (R&D Systems), CD44, and FN1.

### Immunohistochemistry

Immunohistochemistry was conducted using a procedure previously described 50. Osteoblasts on a coverslip were washed briefly in PBS and fixed in 4% paraformaldehyde solution. After blocking using FBS, cells were incubated with the anti-β-catenin (1:200) antibody at 4°C for 24 h, followed by anti-rabbit IgG (1:200) at room temperature for 1 h. Staining with DAB (3,3'-Diaminobenzidine) was conducted and cell images were captured by an optical microscope with 20X magnification at 5 locations (top, bottom, left, right, and center) per coverslip. Cells with a brown-stained spot for β-catenin expression were identified as positive, and the number of positive cells, as well as the total number of cells, was counted in a blinded fashion.

### Plasmid and siRNA transfection

For overexpressing Lrp5 (#115907, Addgene, Watertown, MA, USA), β-catenin (#31785, Addgene), and OPN (#106449, Addgene), each of their plasmids was transfected to MC3T3 osteoblasts or EO771 mammary tumor cells, while a blank plasmid vector (FLAG-HA-pcDNA3.1; Addgene) was used as control. RNA interference was conducted using siRNA specific to Lrp5, CD44, and FN1 (s69315, s63659, s66182; Thermo-Fisher) with a negative siRNA (Silencer Select #1, Thermo-Fisher) as a nonspecific control, using the procedure previously described 47.

### Differentiation of osteoclasts and osteoblasts

The differentiation of RAW264.7 pre-osteoclasts was performed in a 12-well plate in response to 40 ng/mL of RANKL. During the 6-day incubation, the culture medium was exchanged once on day 4. Adherent cells were fixed and stained with a tartrate-resistant acid phosphate (TRAP)-staining kit (Sigma), according to the manufacturer's instructions. TRAP-positive multinucleated cells (> 3 nuclei) were identified as mature osteoclasts. To evaluate the effect of Lrp5 CM on the differentiation of osteoblasts, MC3T3 osteoblasts were cultured in the osteogenic medium that consisted of 50 μg/mL ascorbic acid and 10 mM sodium β-glycerophosphate with 10% FBS and antibiotics. The medium was exchanged every 3 days and cells were fixed and stained with Alizarin Red to visualize calcium deposits in 4 weeks.

### Tumor selectivity assay

Using the MTT-based metabolic activity, we defined tumor selectivity, a parameter defined as a ratio of (reduction in MTT-based metabolic activity of tumor cells) to (reduction in MTT-based activity of non-tumor cells). The tumor selectivity should be above one to preferentially inhibit the metabolic activity of tumor cells and not non-tumor cells. The tumor selectivity was determined using four tumor lines (EO771 mammary tumor cells, MDA-MB-231 breast cancer cells, and PC-3 and TRAMP prostate cancer cells) and four non-tumor cell types (MC3T3 osteoblasts, adipose-derived human MSCs, MLO-A5 osteocytes, and murine MSCs).

### *Ex vivo* cancer tissue assay

The usage of human breast cancer and prostate cancer tissues was approved by the Indiana University Institutional Review Board and the tissues were received from Simon Cancer Center Tissue Procurement Core. A sample (~ 1 g) was manually fragmented with a scalpel into small pieces (0.5 ~ 0.8 mm in length), which were grown in DMEM with 10% fetal bovine serum and antibiotics for a day. Lrp5 CM or BML284-treated CM was then given for two additional days, and a change in the fragment size was blindly determined.

### Animal models

The experimental procedures using animals were approved by the Indiana University Animal Care and Use Committee and were complied with the Guiding Principles in the Care and Use of Animals endorsed by the American Physiological Society. Mice were housed five per cage and provided with mouse chow and water *ad libitum*. They were randomly assigned into the placebo and treatment groups. In the mouse model of a mammary tumor, C57BL/6 female mice (10 mice per group, ~8 weeks, Envigo RMS, Inc., Indianapolis, IN, USA) received subcutaneous injections of EO771 cells (3.0 × 10^5^ cells in 50 μL PBS) to the seventh mammary fat pad on day 1. From day 2, mice received a daily intravenous injection of Lrp5 CM. The animals were sacrificed on day 18, and the weight of each tumor was measured.

In the mouse model of osteolysis, ten C57BL/6 male and female mice per group received an injection of EO771 and TRAMP cells (3.0 × 10^5^ cells in 20 μL PBS), respectively, into the right tibia as an intra-tibial injection. Lrp5 CM was given daily as an intravenous injection to the tail vein. To visualize tumor-colonized bone, we additionally employed NOD/SCID/γ(-/-) (NSG) female mice (placebo and Lrp5 CM groups, 5 mice per group; *In vivo* Therapeutics Core of the Indiana University Simon Cancer Center, Indianapolis, IN, USA). NSG mice received GFP-labeled MDA-MB-231 breast cancer cells (2.5 × 10^5^ cells in 20 μL PBS) as an intra-tibial injection. Mice were sacrificed in 18 days and the tibiae were harvested for histology. Tibia samples were decalcified, dehydrated through a series of graded alcohols, cleared in xylene, and embedded in paraffin. To determine the distribution of tumor cells, we evaluated the sections from the proximal tibia at 60-μm intervals. Images were taken from 5 locations per slide, and the tumor area was quantified as a ratio of the tumor-colonized area to the total area in a blinded fashion.

### Lung metastasis and hiotology

To evaluate the effect of Lrp5 CM on tumor invasion to the lung, EO771 tumor cells (~ 3.0 × 10^5^ cells in 100 μL PBS) were inoculated to C57BL/6 mice (placebo and Lrp5 CM groups, 10 mice per group) as an intracardiac injection. The placebo mice received a daily i.v. injection of PBS, while the mice in the treatment group received Lrp5 CM. To visualize tumor-invaded lungs, we additionally inoculated GFP-labeled MDA-MB-231 breast cancer cells (~ 1.0 × 10^5^ cells in 100 μL PBS) to NSG female mice (placebo and Lrp5 CM groups, 5 mice per group) as an i.v. injection from the tail vein. The Lrp5 CM group received a daily injection of Lrp5 CM as a tail-vein injection from day 2 to 14. C57BL/6 and NSG mice were sacrificed in 3 and 2 weeks, respectively, and the presence of tumor cells in the lung was determined histologically. Lung samples were harvested and dehydrated through a series of graded alcohols, cleared in xylene, and embedded in paraffin. The samples were sectioned at 60-μm intervals. Images were taken from 5 locations per slide, and the tumor area was quantified as a ratio of the tumor-colonized area to the total area in a blinded fashion.

### microCT imaging and histology

Micro-computed tomography was performed with the tibiae of C57BL/6 mice using Skyscan 1172 (Bruker-MicroCT, Kontich, Belgium). Using manufacturer-provided software, scans were performed at pixel size 8.99 μm and the images were reconstructed (nRecon v1.6.9.18) and analyzed (CTan v1.13). The region of interest was 1 mm in length along the length of the tibia, from the cross-section immediately distal to the growth plate. We determined four trabecular bone parameters such as bone volume ratio (BV/TV), bone mineral density (BMD), trabecular number (Tb.N), and trabecular separation (Tb.Sp) in a blinded fashion. In histology, H&E staining was conducted as described previously 51. Of note, normal bone cells appeared in a regular shape with round and deeply stained nuclei, while tumor cells were in a distorted shape with irregularly stained nuclei.

### Statistical analysis

For cell-based experiments, three or four independent experiments were conducted and data were expressed as mean ± S.D. In animal experiments, the sample size in the mouse model was chosen to achieve a power of 80% with *p* < 0.05. The primary experimental outcome was tumor weight for the mammary fat pad experiment and the bone volume ratio (BV/TV) for the tibia experiment. The secondary experimental outcome was tumor size for the mammary fat pad experiment and the trabecular number (Tb.n) for the tibia experiment. Statistical significance was evaluated using a one-way analysis of variance (ANOVA). Post hoc statistical comparesons with control groups were performed using Bonferroni correction with statistical significance at *p* < 0.05. The single and double asterisks in the figures indicate *p* < 0.05 and *p* < 0.01, respectively.

## Supplementary Material

Supplementary figures.

## Abbreviations

ALP: alkaline phosphatase
BMD: bone mineral density
BV/TV: bone volume ratio
CM: conditioned medium
CXCL12: C-X-C motif ligand 12
DMEM: Dulbecco's modified Eagle's medium
ECM: extracellular matrix
EdU: 5-ethynyl-2'-deoxyuridine
EGF: epidermal growth factor
FN1: fibronectin 1
H&E: hematoxylin and eosin
Hsp90ab1: heat shock protein 90 alpha class B, member 1
iTS cells: induced tumor-suppressing cells
KDM3A: lysine demethylase 3A
Lrp5: low-density lipoprotein receptor-related protein 5
M-CSF: macrophage colony-stimulating factor
MMP9: matrix metalloproteinase 9
MSCs: mesenchymal stem cells
MSN: moesin
OPN: osteopontin
OPG: osteoprotegerin
PDL-1: programmed death ligand-1
Runx2: runt-related transcription factor 2
Tb.N: trabecular number
Tb.Sp: trabecular separation
TGFβ: transforming growth factor beta
