# Suppression of breast cancer-associated bone loss with osteoblast proteomes via Hsp90ab1/moesin-mediated inhibition of TGFβ/FN1/CD44 signaling: Erratum

**DOI:** 10.7150/thno.79085

**Published:** 2023-01-01

**Authors:** Xun Sun, Kexin Li, Misato Hase, Rongrong Zha, Yan Feng, Bai-Yan Li, Hiroki Yokota

**Affiliations:** 1 Department of Pharmacology, School of Pharmacy, Harbin Medical University, Harbin 150081, China; 2 Department of Biomedical Engineering, Indiana University Purdue University Indianapolis, Indianapolis, IN 46202, USA; 3 Graduate School of Engineering, Mie University, Mie 514, Japan; 4 Indiana Center for Musculoskeletal Health, Indiana University School of Medicine, Indianapolis, IN 46202, USA; 5 Simon Cancer Center, Indiana University School of Medicine, Indianapolis, IN 46202, USA

The authors regret that the original version of our paper, unfortunately, contained three incorrect images in Figure 1H, Figure 4C, and Figure S3B. The correct version is shown below.

The correction made in this erratum does not affect the original data and conclusions. The authors apologize for any inconvenience that the errors may have caused.

## Figures and Tables

**Figure 1:**
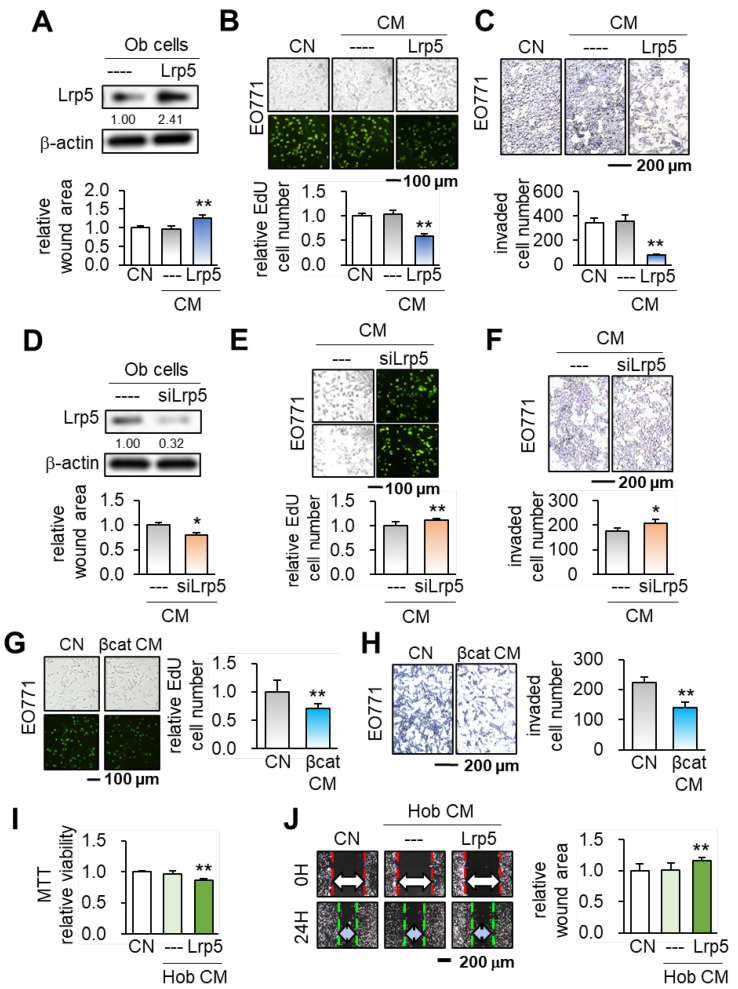
Suppression of the tumorigenic behaviors of EO771 mammary tumor cells by MC3T3 osteoblasts with the overexpression of Lrp5 and β-catenin, and the treatment of BML284. Ob = MC3T3 osteoblasts, Hob = human osteoblasts, CM = osteoblast-derived conditioned medium, siL5 = Lrp5 siRNA, and βcat = β-catenin overexpression. The single and double asterisks indicate p < 0.05 and 0.01, respectively. **(A-C)** Reduction in the scratch-based motility, EdU-based proliferation, and transwell invasion of EO771 mammary tumor cells by Lrp5 CM. **(D-F)** Elevation in the scratch-based motility, EdU-based proliferation, and transwell invasion by Lrp5-silenced CM. **(G-H)** Reduction in the EdU-based proliferation and transwell invasion of EO771 mammary tumor cells by β-catenin Ob CM. **(I-J)** Reduction in the MTT-based proliferation and scratch-based motility of MDA-MB-231 breast cancer cells by Lrp5-overexpressing Hob CM.

**Figure 4:**
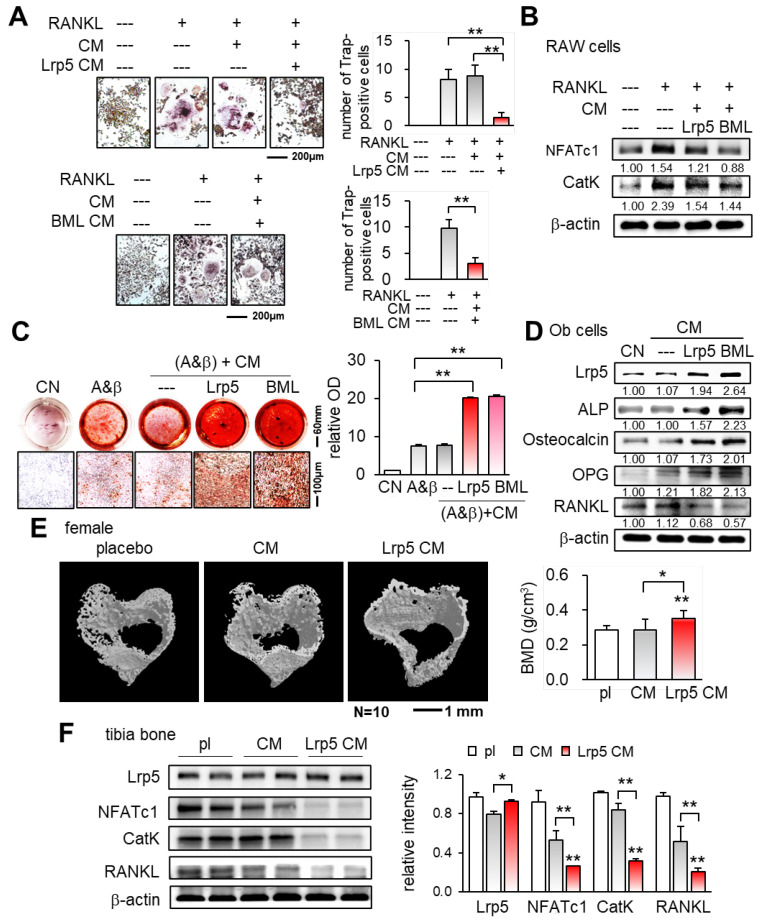
Suppression of osteoclast differentiation and stimulation of osteoblast differentiation by Lrp5 CM and BML284-treated CM. Ob = osteoblasts, CM = osteoblast-derived conditioned medium, A&β = ascorbic acid and β-glycerophosphate, pl = placebo, and Lrp5 = Lrp5 overexpression. The single and double asterisks indicate p < 0.05 and 0.01, respectively. **(A)** Trap staining of RANKL-stimulated RAW264.7 pre-osteoclasts in response to Ob CM, Lrp5 CM, and BML284-treated CM. **(B)** Downregulation of NFATc1 and cathepsin K by Lrp5 CM and BML284-treated CM. **(C)** Enhanced Alizarin-red staining of MC3T3 osteoblasts by Lrp5 CM and BML284-treated CM. **(D)** Elevation of Lrp5, ALP, osteocalcin, and OPG, and the reduction of RANKL by Lrp5 CM and BML284-treated CM in MC3T3 osteoblasts. **(E)** Effect of Lrp5 CM on tibial cortical bone. BMD = bone mineral density, N = 10. **(F)** Downregulation of NFATc1, cathepsin K, and RANKL in the tibia by Lrp5 CM.

**Figure S3:**
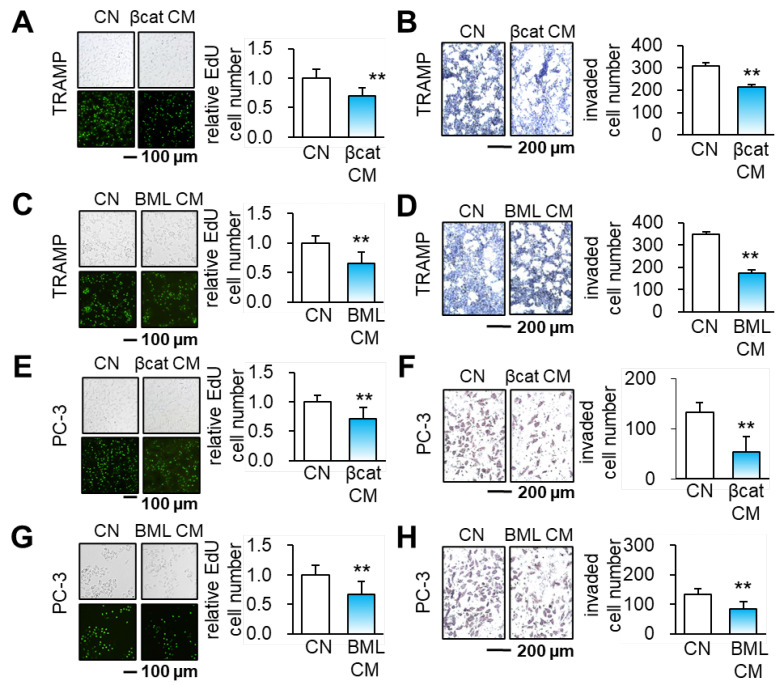
Reduction in the EdU-based proliferation, and transwell invasion of TRAMP and PC-3 prostate tumor cells by β-catenin CM and BML284-treated CM. **(A-B)** TRAMP prostate tumor cells in response to β-catenin CM. **(C-D)** TRAMP cells in response to BML284-treated CM. **(E-F)** PC-3 prostate cancer cells in response to β-catenin CM. **(G-H)** PC-3 cells in response to BML284-treated CM.

