# Supplementary material for: Suppression of breast cancer-associated bone loss with osteoblast proteomes via Hsp90ab1/moesin-mediated inhibition of TGFβ/FN1/CD44 signaling
Source: Theranostics. 2022 Jan 1;12(2):929–43. doi: 10.7150/thno.66148 (PMC8692912; doi:10.7150/thno.66148)
Supplement: Supplementary file 1 — Supplementary figures. [file thnov12p0929s1.pdf]

Supplementary materials

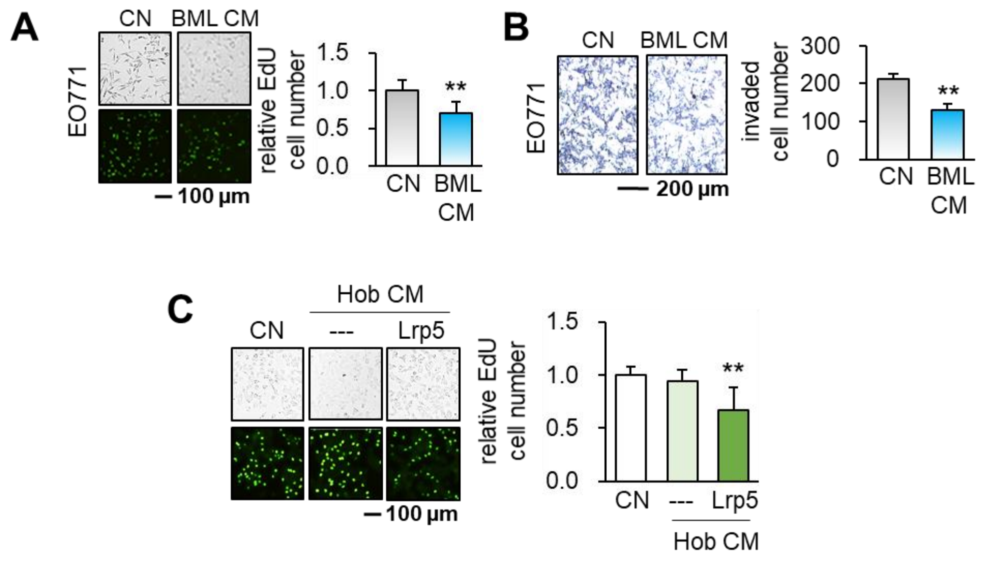

Figure S1. Suppression of the tumorigenic behaviors of EO771 mammary tumor cells and MDA-MB-231 breast cancer cells by the conditioned media that were derived from MC3T3 osteoblasts and human Hob osteoblasts. MC3T3 cells were treatment with 0.3 μM of BML284, while Hob cells were overexpressed with Lrp5. The double asterisk indicates $p < 0.01$. CN = control, CM = conditioned medium, and Ob = osteoblast. **(A-B)** Reduction in EdU-based proliferation and transwell invasion by BML284-treated Ob CM. **(C)** Reduction in EdU-based proliferation of MDA-MB-231 breast cancer cells by Lrp5-overexpressed Hob CM.

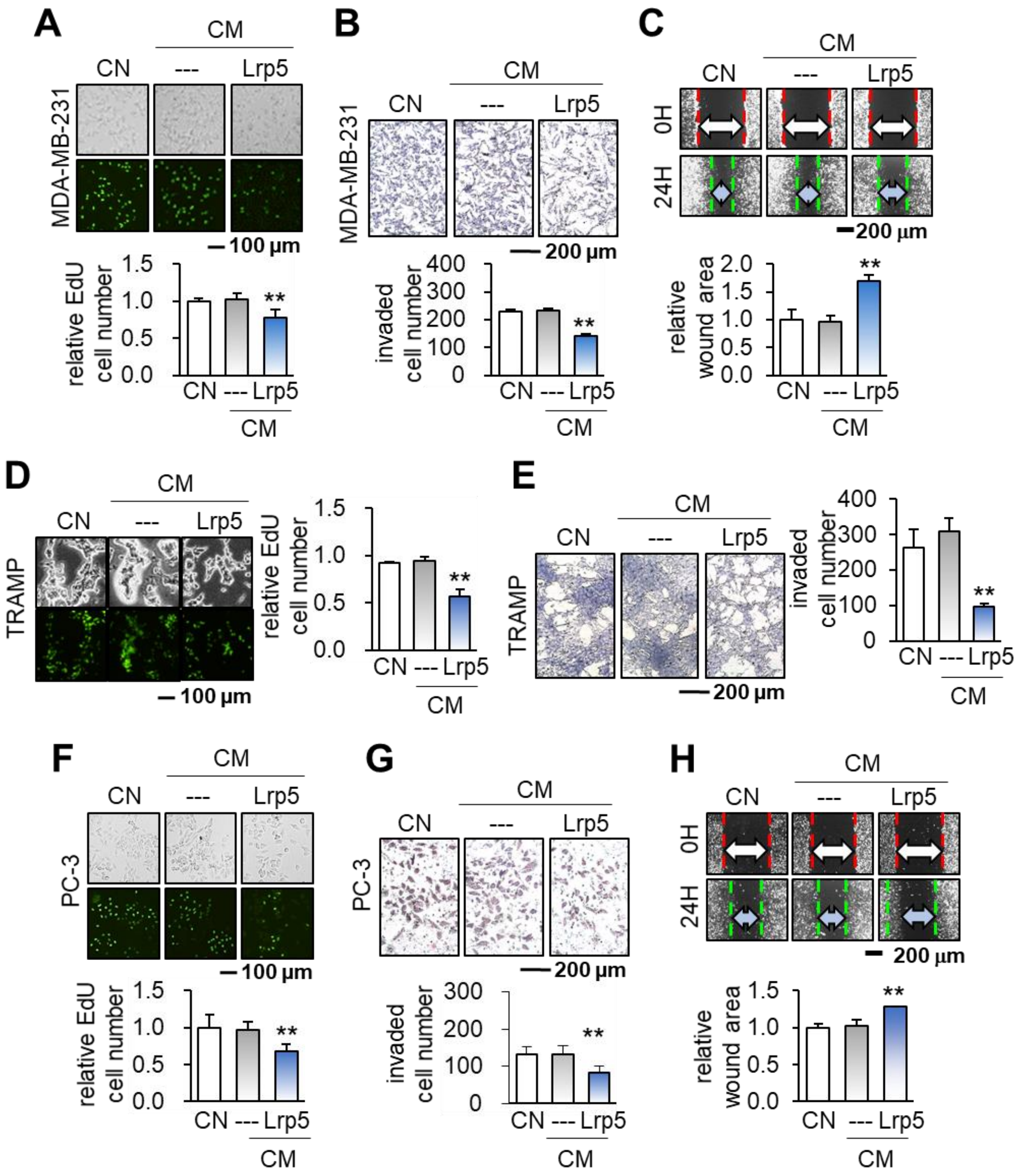

Figure S2. Suppression of tumorigenic behaviors of MDA-MB-231 breast cancer cells as well as TRAMP and PC-3 prostate cancer cells by Lrp5 CM. The double asterisk indicates $p < 0.01$. **(A-C)** Reduction in the EdU-based proliferation, transwell invasion, and scratch-based migration of MDA-MB-231 breast cancer cells by Lrp5 CM. **(D-E)** Reduction in the EdU-based proliferation and transwell invasion of TRAMP prostate tumor cells by Lrp5 CM. **(F-H)** Reduction in the MTT-based viability, transwell invasion, and scratch-based motility of PC-3 prostate cancer cells by Lrp5 CM.

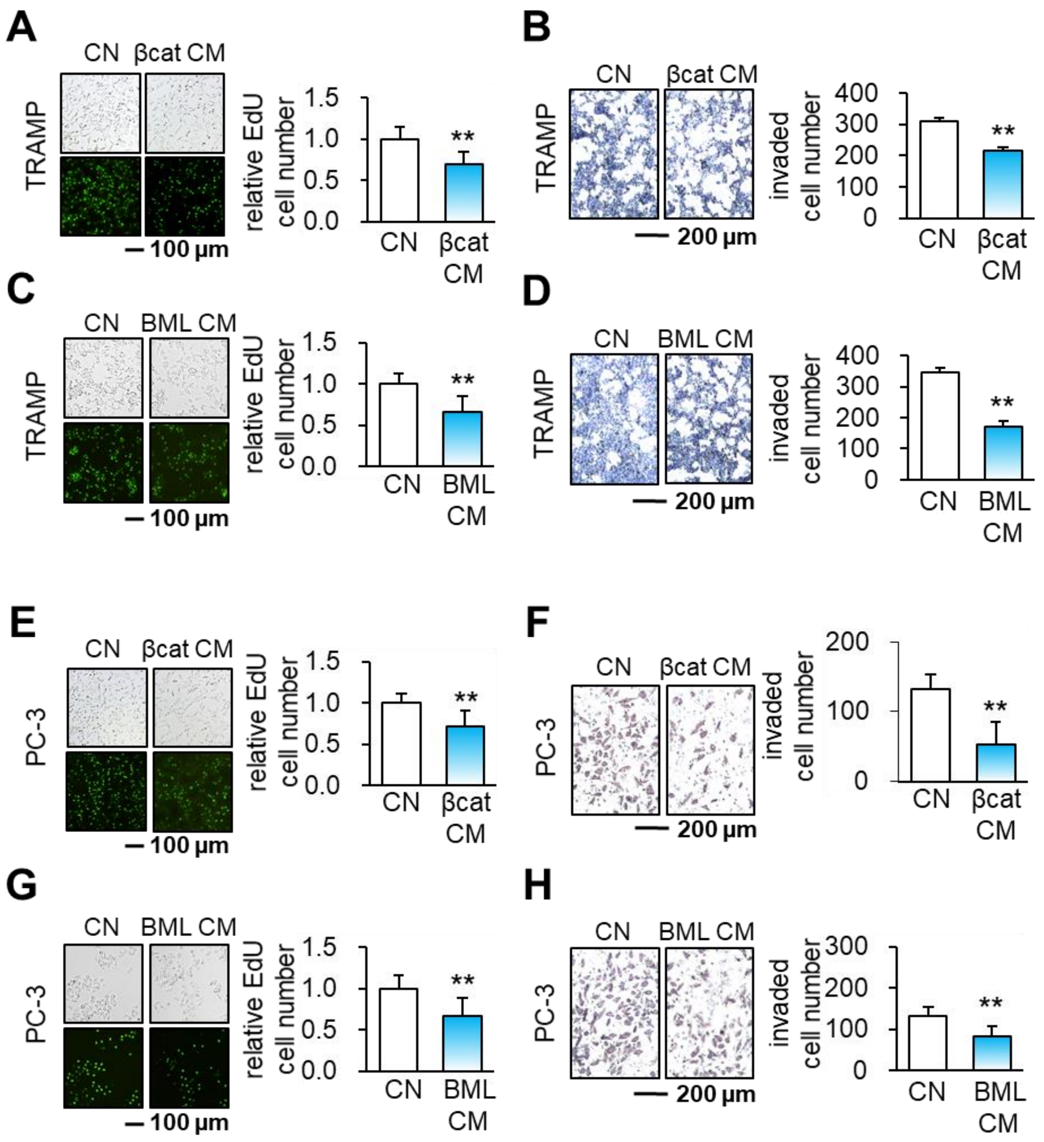

Figure S3. Reduction in the EdU-based proliferation, and transwell invasion of TRAMP and PC-3 prostate tumor cells by β-catenin CM and BML284-treated CM. **(A-B)** TRAMP prostate tumor cells in response to β-catenin CM. **(C-D)** TRAMP cells in response to BML284-treated CM. **(E-F)** PC-3 prostate cancer cells in response to β-catenin CM. **(G-H)** PC-3 cells in response to BML284-treated CM.

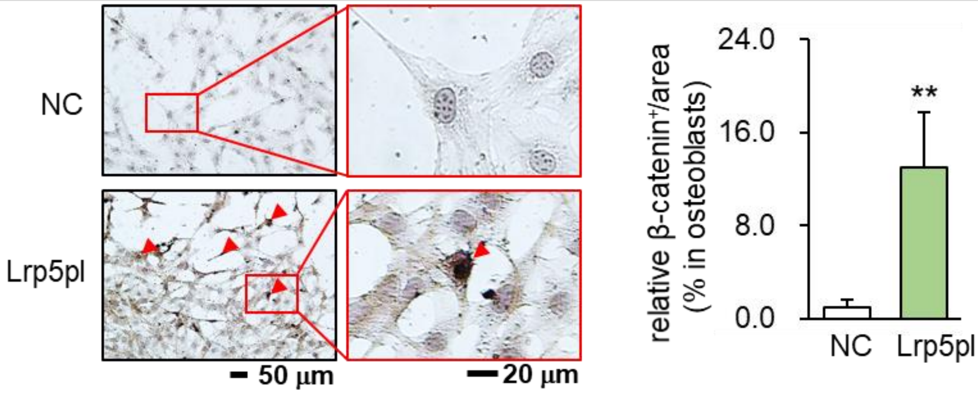

Figure S4. Immunohistochemical analysis of osteoblasts with and without Lrp5 overexpression. The upper images are the negative control (NC), while the lower images are Lrp5-overexpressing cells (Lrp5pl). The red arrowheads indicate the region with β-catenin expression (brown staining).

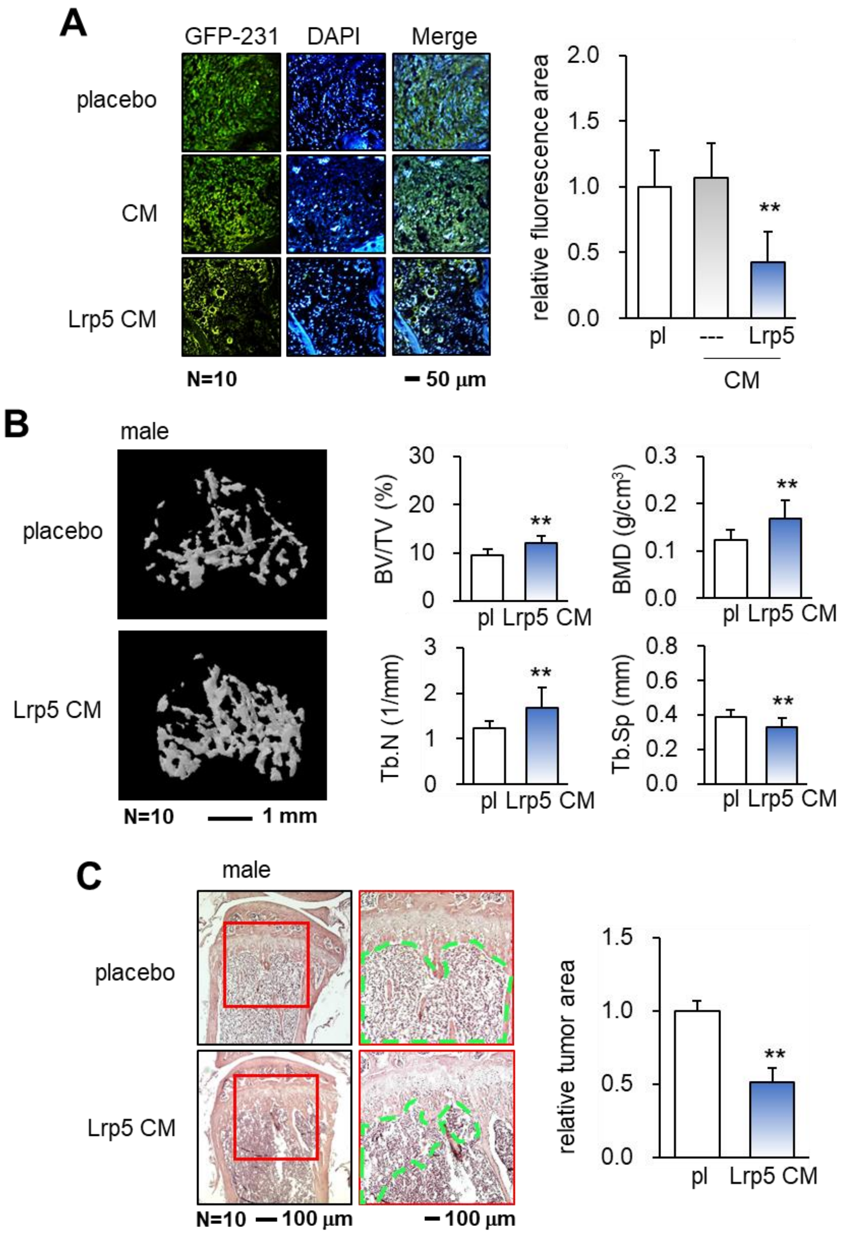

Figure S5. Protection of the prostate tumor-invaded bone of C57BL/6 mice by Lrp5 CM. pl = placebo, Lrp5 = Lrp5 overexpression, and CM = osteoblast-derived conditioned medium. The double asterisk indicates $p < 0.01$. **(A)** Significant reduction in the tumor-invaded area in the tibia of NOD/SCID/γ(-/-) mice in response to Lrp5-overexpressing human osteoblast-derived CM. **(B)** Inhibition of bone loss in the tumor-invaded tibia by Lrp5 CM. BV/TV = bone volume ratio, BMD = bone mineral density, Tb.N = trabecular number, and Tb.Sp = trabecular separation, N = 10. **(C)** Reduction in the tumor-invaded area (green-outlined region) by the daily administration of Lrp5 CM, N = 10.

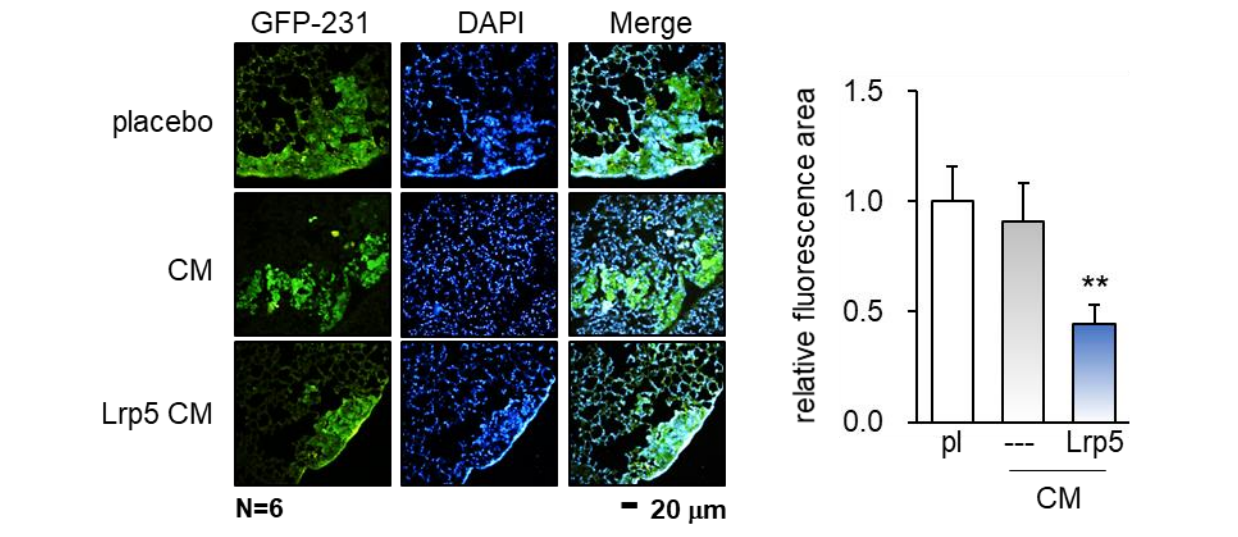

Figure S6. Significant reduction in the tumor-invaded area in the lung of NOD/SCID/γ(-/-) mice in response to Lrp5-overexpressing human osteoblast-derived CM.

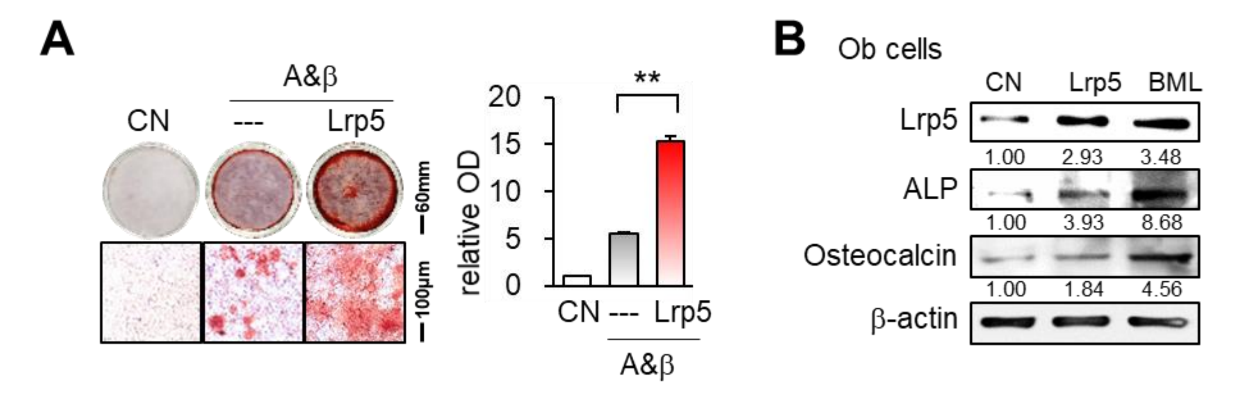

Figure S7. Stimulation of osteoblast differentiation by the overexpression of Lrp5 and BML284 treatment. A = ascorbic acid, β = β-glycerophosphate, Lrp5 = Lrp5 overexpression, and Ob = osteoblasts. The double asterisk indicates $p < 0.01$. **(A)** Enhanced Alizarin-red staining of MC3T3 osteoblasts by the overexpression of Lrp5. **(B)** Elevation of Lrp5, ALP (alkaline phosphatase), and osteocalcin by Lrp5 overexpression and BML284 treatment.

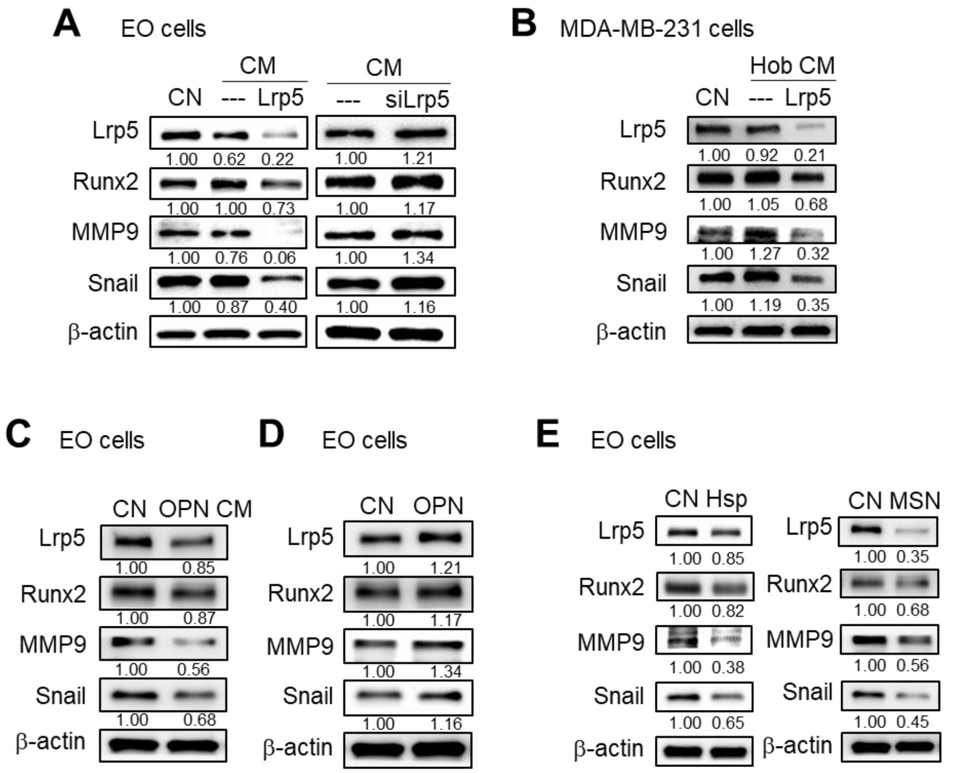

Figure S8. Tumor-suppressing effects of Lrp5- overexpressing Ob CM, Hsp90ab1, and MSN, and dichotomous role of osteopontin in EO771 cells. CN = control, EO = EO771 mammary tumor cells, Hob = human osteoblasts, CM = osteoblast-derived conditioned medium, Lrp5 = Lrp5 plasmid, siLrp5 = Lrp5 siRNA, OPN = osteopontin plasmid, Hsp = Hsp90ab1, and MSN = moesin. **(A)** Downregulation of Lrp5, Runx2, MMP9, and Snail in EO771 mammary tumor cells by Lrp5 CM and the elevation by Lrp5-silenced CM. **(B)** Downregulation of Lrp5, Runx2, MMP9, and Snail in MDA-MB-231 mammary tumor cells in response to Lrp5-overexpressing Hob CM. **(C)** Downregulation of Lrp5, Runx2, MMP9, and Snail in EO771 cells by OPN-overexpressing CM. **(D)** Elevation of Lrp5, Runx2, MMP9, and Snail in EO771 cells by the overexpression of OPN. **(E)** Downregulation of Lrp5, Runx2, MMP9, and Snail in EO771 cells by the application of 5 μg/mL Hsp90ab1 and MSN.

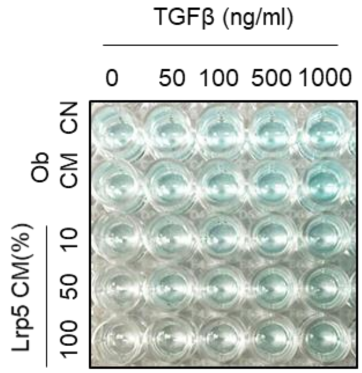

Figure S9. Cell ELISA-based stained levels of PDL-1 expression in EO771 tumor cells in response to the gradient of TGFβ and Lrp5 CM. CN = control, Ob = osteoblasts, L5 = overexpression of Lrp5, and CM = conditioned medium.

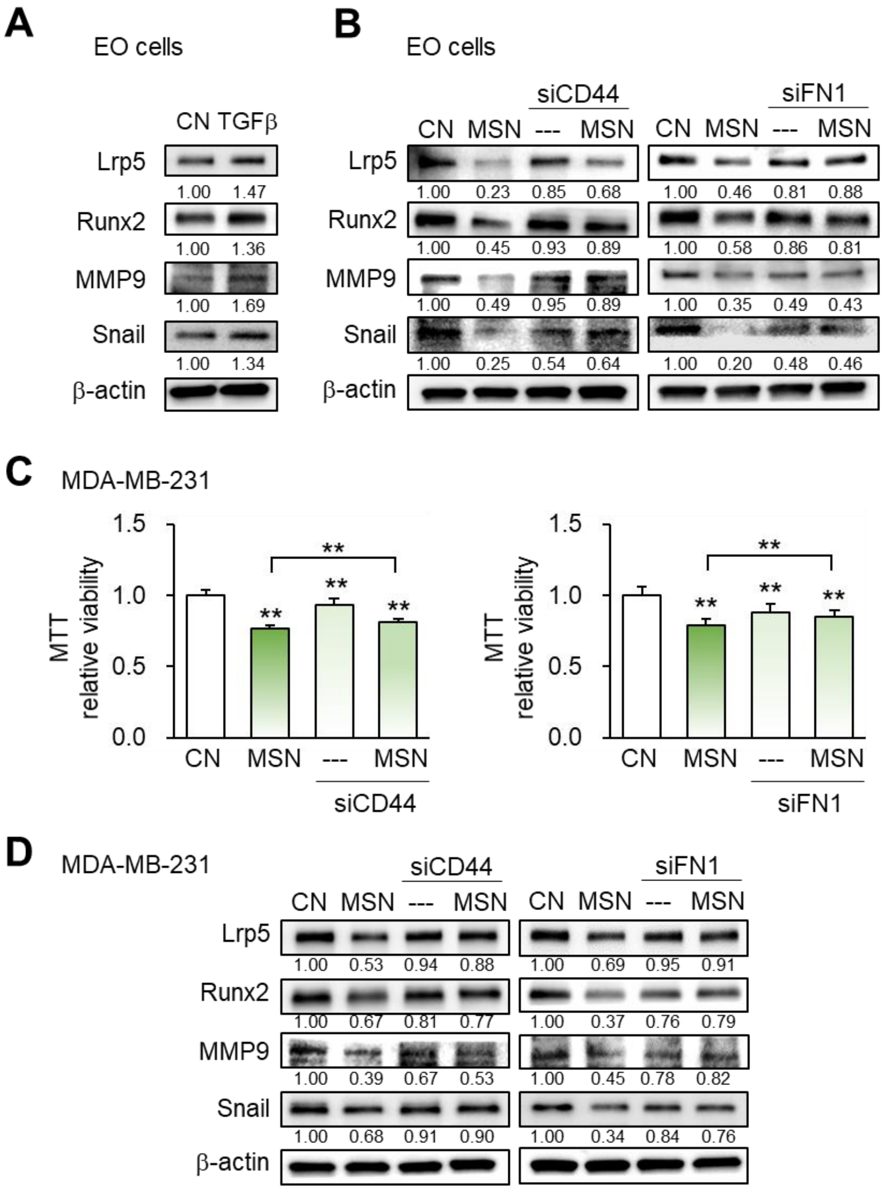

Figure S10. Tumor-promoting effect of TGFβ recombinant proteins, and the anti-tumor effect by silencing CD44 and FN1 in EO771 cells. **(A)** Elevation of Lrp5, Runx2, MMP9, and Snail in EO771 cells by the application of 750 ng/mL TGFβ. **(B)** Suppression of MSN-mediated downregulation of Lrp5, MMP9, Runx2, and Snail in EO771 cells by RNA silencing of CD44 and FN1. **(C)** Suppression of MSN-mediated inhibition of the proliferation of MDA-MB-231 cells by RNA silencing of CD44 and FN1. **(D)** Suppression of MSN-mediated downregulation of Lrp5, MMP9, Runx2, and Snail in MDA-MB-231 cells by RNA silencing of CD44 and FN1.

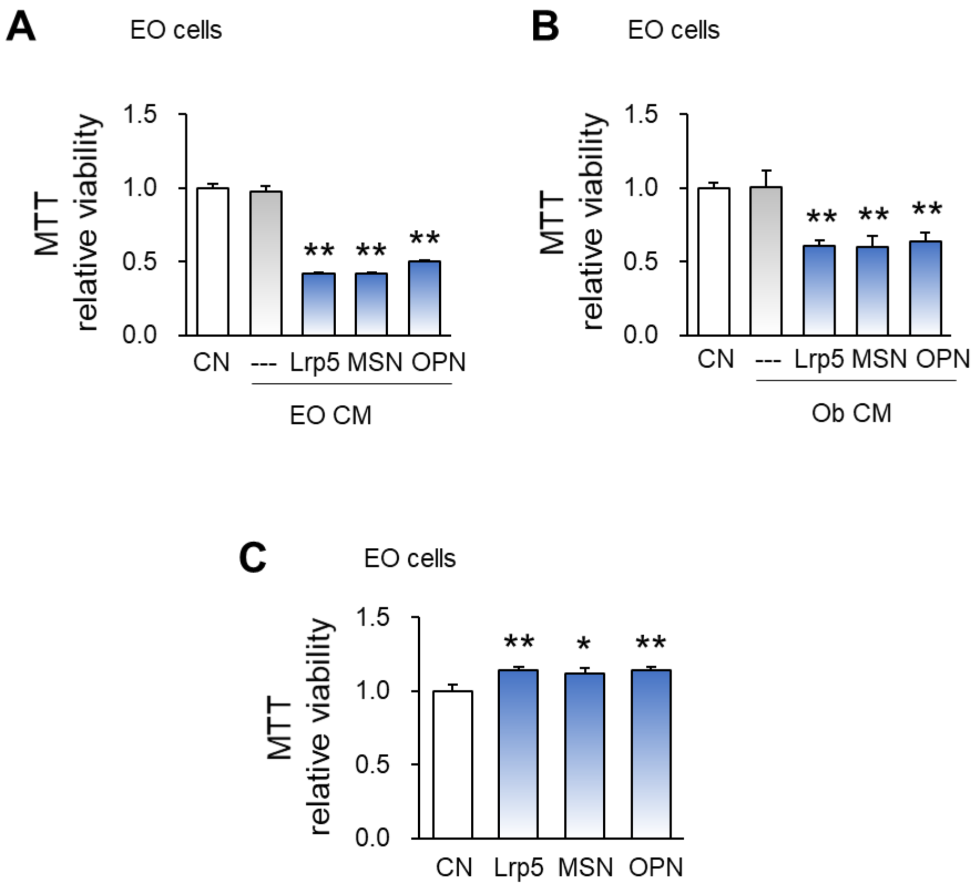

Figure S11. Dichotomous role of Lrp5, MSN, and osteopontin in osteoblasts and EO771 mammary tumor cells. CN = control, EO = EO771, Ob = osteoblasts, Lrp5 = overexpression of Lrp5, OPN = overexpression of osteopontin, MSN = overexpression of moesin, and CM = conditioned medium. The single and double asterisks indicate $p < 0.05$ and 0.01, respectively. **(A)** Reduction in the MTT-based viability by Lrp5-, MSN-, and Osteopontin-overexpressing EO771 tumor cell-derived CMs. **(B)** Reduction in the MTT-based viability by Lrp5-, MSN-, and osteopontin-overexpressing osteoblast-derived CMs. **(C)** Elevation of the MTT-based viability of EO771 tumor cells by the overexpression of Lrp5, MSN, and OPN.
